# Deficiency of Perry syndrome-associated p150^Glued^ in midbrain dopaminergic neurons leads to progressive neurodegeneration and endoplasmic reticulum abnormalities

**DOI:** 10.1038/s41531-023-00478-0

**Published:** 2023-03-07

**Authors:** Jia Yu, Xuan Yang, Jiayin Zheng, Carmelo Sgobio, Lixin Sun, Huaibin Cai

**Affiliations:** 1 Basic Research Center, Institute for Geriatrics and Rehabilitation, Beijing Geriatric Hospital, Beijing, 100095 China; 2 Transgenics Section, Laboratory of Neurogenetics, National Institute on Aging, National Institutes of Health, Bethesda, MD 20892 USA; 3 Center for Neuropathology and Prion Research, Ludwig-Maximilians University Munich, Munich, 81377 Germany

**Keywords:** Parkinson's disease, Parkinson's disease

## Abstract

Multiple missense mutations in p150^Glued^ are linked to Perry syndrome (PS), a rare neurodegenerative disease pathologically characterized by loss of nigral dopaminergic (DAergic) neurons. Here we generated p150^Glued^ conditional knockout (cKO) mice by deleting p150^Glued^ in midbrain DAergic neurons. The young cKO mice displayed impaired motor coordination, dystrophic DAergic dendrites, swollen axon terminals, reduced striatal dopamine transporter (DAT), and dysregulated dopamine transmission. The aged cKO mice showed loss of DAergic neurons and axons, somatic accumulation of α-synuclein, and astrogliosis. Further mechanistic studies revealed that p150^Glued^ deficiency in DAergic neurons led to the reorganization of endoplasmic reticulum (ER) in dystrophic dendrites, upregulation of ER tubule-shaping protein reticulon 3, accumulation of DAT in reorganized ERs, dysfunction of COPII-mediated ER export, activation of unfolded protein response, and exacerbation of ER stress-induced cell death. Our findings demonstrate the importance of p150^Glued^ in controlling the structure and function of ER, which is critical for the survival and function of midbrain DAergic neurons in PS.

## Introduction

Motor protein dynein and its activator, dynactin, move along the microtubule towards the minus end, playing crucial roles in mitosis and intracellular transport^1–4^. Genetic mutations in genes encoding components of dynein and dynactin are associated with various neurological diseases^5–7^. The G59S missense mutation in dynactin subunit p150^Glued^, encoded by the *DCTN1* gene, has been linked to an autosomal dominant motor neuron disease (MND)^8^. Since then, another 13 missense mutations (F52L, K56R, G67D, K68E, G71A/R/E/V, T72P, Q74P, Y78C/H, and Q93H) in p150^Glued^ have been identified as the genetic cause of Perry syndrome (PS), an autosomal dominant neurodegenerative disease characterized by parkinsonism with mental depression, weight loss, and central hypoventilation^9–13^. Severe loss of dopaminergic (DAergic) neurons in the *substantia nigra* (SN) and dysfunction of dopamine (DA) transmission in the striatum were reported in PS patients^10–16^. However, the pathogenic mechanism underlying the degeneration of midbrain DAergic neurons in PS remains elusive.

P150^Glued^ contains tandem microtubule-binding domains (MTBDs) at its N-terminus: the cytoskeleton-associated protein and glycine-rich (CAP-Gly) domain and the basic domain^17–19^. The CAP-Gly domain has the binding affinity for microtubules and microtubule end-binding proteins^17–19^. The *DCTN1* gene, composed of 32 exons, not only synthesizes the MTBDs-containing p150^Glued^ but also encodes p135 and other short splicing isoforms, collectively called p135+, which lack the MTBDs^18–22^. The MTBDs in p150^Glued^ possess neuron-specific functions, such as facilitating the initiation of retrograde transport from the distal axons and enhancing the stability of microtubules along axons^23–25^. The MND- and PS-related mutations in p150^Glued^ occur within or close to the CAP-Gly domain, disrupt the association of p150^Glued^ with microtubules and microtubule end-binding proteins, and thereby inhibit the initiation of retrograde transport and destabilize microtubules in axons^8–13,23–26^. These findings indicate a critical role of MTBDs in the pathogenesis of neurodegeneration induced by the mutations of p150^Glued^.

Knock-in (KI) and transgenic mice expressing MND-related G59S mutant p150^Glued^, as well as the *Dctn1*^LoxP/LoxP^;*Thy1-Cre* mice which lack the MTBDs-containing p150^Glued^ but express p135+ in the forebrain and spinal neurons, have been generated^22,27–29^. Studies on these mouse models reveal multiple subcellular abnormalities in spinal motor neurons which might contribute to the neurodegeneration induced by the mutation or lack of MTBDs in p150^Glued^, including impaired distal axonal integrity, and retrograde axonal transport, aberrant endoplasmic reticulum (ER)-Golgi secretory pathway and autophagosome-lysosome degradative pathway, and augmentations of postsynaptic glutamate receptors and susceptibility to excitotoxicity^22,27–29^. While KI and transgenic mice expressing PS-related mutant p150^Glued^ have generated and recapitulated some of PS’s clinical and pathological features^30–32^, the vital intracellular pathways and the underlying molecular mechanisms responsible for the selective degeneration of midbrain DAergic neurons induced by the mutation or lack of MTBDs in p150^Glued^ remain to be determined.

In this study, we investigated the importance of p150^Glued^ and its MTBDs in the survival and function of midbrain DAergic neurons using genetically engineered mouse models. Through crossbreeding *Dctn1*^LoxP/^ mice^22^ and *Th-Cre* transgenic mice^33^, we generated *Dctn1* ^LoxP/LoxP^;*Th-Cre* conditional knockout (cKO) mice, which lacked the MTBDs-containing p150^Glued^ but expressed p135+ in midbrain DAergic neurons. We then performed a series of in vivo and in vitro experiments to identify behavioral, neuropathological, electrochemical, subcellular, and molecular abnormalities in the cKO mice. The cKO mice displayed aggravated impairment of motor coordination during aging and progressive degeneration of midbrain DAergic neurons. Furthermore, p150^Glued^ deficiency leads to multiple changes in ER of midbrain DAergic neurons, including the reorganization of ER structure, dysfunction of ER export, activation of unfolded protein response, and increased susceptibility to ER stress-induced cell death, suggesting the abnormal ER structure and function contribute to the DAergic neurodegeneration in PS.

## Results

### Generation of cKO mice with deletion of p150^Glued^ in midbrain DAergic neurons

**Fig. 1:**
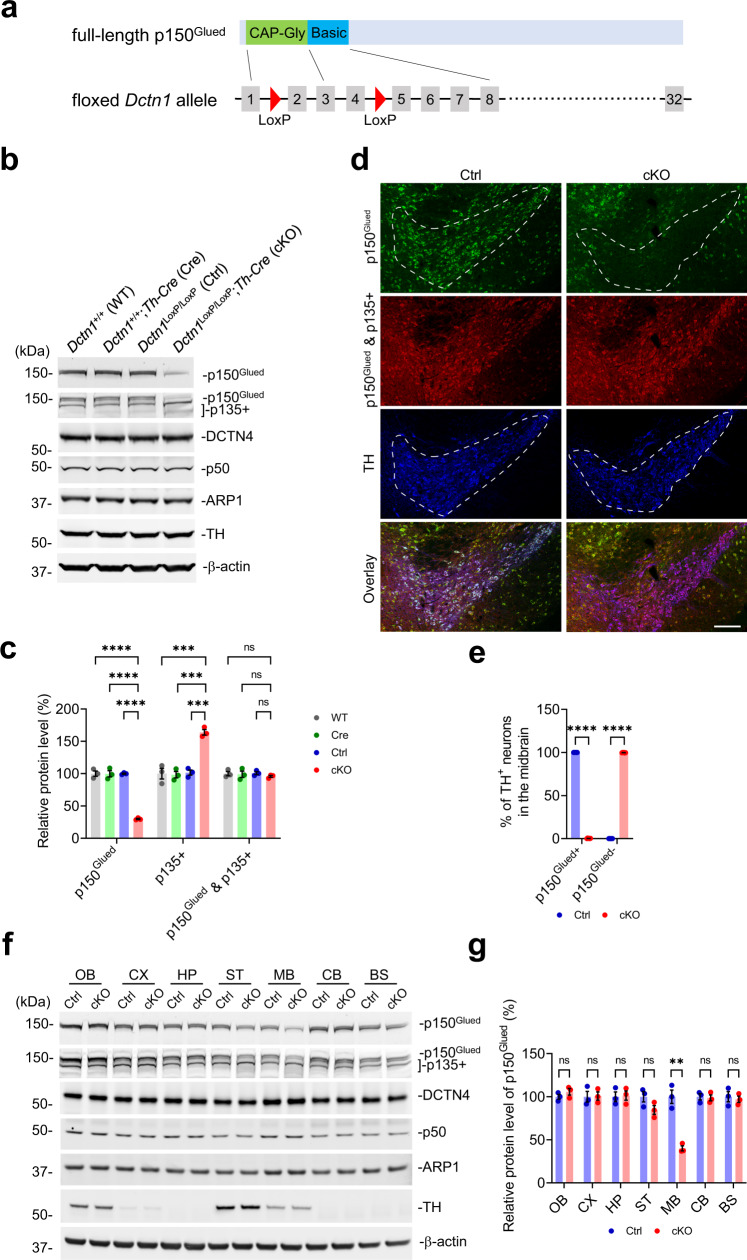
Genetic deletion of p150^Glued^ in midbrain DAergic neurons of cKO mice. **a** The schematic diagram depicts the full-length p150^Glued^ and the floxed *Dctn1* allele of mice. The N-terminal CAP-Gly domain and the adjacent basic domain (encoded by the exon 1–3 and 3–8 of the *Dctn1* gene, respectively) form the tandem MTBDs of p150^Glued^. The floxed *Dctn1* allele has two LoxP sites inserted at the intron 1 and 4 of the *Dctn1* gene locus. **b**, **c** Western blots show the expression of p150^Glued^, p135+, DCTN4, p50, ARP1, and TH in the midbrain of 1-month-old WT (*Dctn1*^+/+^), Cre (*Dctn1*^+/+^;*Th-Cre*), Ctrl (*Dctn1*^LoxP/LoxP^), and cKO (*Dctn1*^LoxP/LoxP^;*Th-Cre*) mice. The protein level was quantified as mean ± SEM (*n* = 3 animals per genotype). One-way ANOVA, ^***^p = 0.0002, ^****^p < 0.0001. **d**, **e** Immunofluorescent images show the staining of p150^Glued^ (green), p150^Glued^ & p135+ (red), and TH (blue) in the midbrain of 1-month-old Ctrl and cKO mice. Scale bar: 250 μm. The percentages of p150^Glued^-positive and p150^Glued^-negative TH^+^ neurons were quantified as mean ± SEM (*n* = 6 animals per genotype and 6 sections per animal). Unpaired *t* test, ^****^p < 0.0001. **f**, **g** Western blots show the expression of p150^Glued^, p135+, DCTN4, p50, ARP1, and TH in the olfactory bulb (OB), cortex (CX), hippocampus (HP), striatum (ST), midbrain (MB), cerebellum (CB), and brain-stem (BS) of 1-month-old Ctrl and cKO mice. The protein level was quantified as mean ± SEM (*n* = 3 animals per genotype). Unpaired *t* test, ^**^p = 0.0019.

In our previous work, we generated *Dctn1*^LoxP/^ mice, which had LoxP sites flanking exon 2–4 of the *Dctn1* gene (Fig. 1a)^22^. Cre recombinase-mediated deletion of exon 2–4 from the floxed *Dctn1* abolishes the expression of the MTBDs-containing p150^Glued^ but keeps the expression of the MTBDs-lacking p135+ in the Cre-expressing cells of *Dctn1*^LoxP/LoxP^;*Cre* mice^22^. In the current study, to delete p150^Glued^ in midbrain DAergic neurons, we crossbred *Dctn1*^LoxP/^ mice with *Th-Cre* mice^33^. We obtained *Dctn1*^+/+^ [referred to as wild-type (WT)], *Dctn1*^+/+^;*Th-Cre* (referred to as Cre), *Dctn1*^Loxp/LoxP^ [referred to as control (Ctrl)], and *Dctn1*^LoxP/LoxP^;*Th-Cre* (referred to as cKO) mice. The composition of offspring with different genotypes followed the Mendelian ratio, indicating a normal embryonic development of cKO mice. To examine the selective depletion of p150^Glued^ in cKO mice, we used the antibody specific for the N-terminus of p150^Glued^ (recognizing only p150^Glued^ but not p135+) and the antibody specific for the C-terminus of p150^Glued^ (recognizing both p150^Glued^ and p135+). Western blotting revealed a substantial decrease (approximately 70%) of p150^Glued^ but a marked increase (about 64%) of p135+ in the midbrain tissues dissected from 1-month-old cKO mice compared to the age-matched WT, Cre, and Ctrl mice (Fig. 1b, c). On the other hand, the levels of all the protein isoforms of the *Dctn1* gene (p150^Glued^ & p135+) and other dynactin subunits (including DCTN4, p50, and ARP1) were not significantly changed in the cKO mice (Fig. 1b). The residual p150^Glued^ protein detected in the cKO samples is likely derived from the non-DAergic neurons and glia in the tissue preparations (Fig. 1b, c). Indeed, immunohistochemistry of midbrain sections from 1-month-old cKO mice demonstrated a complete loss of p150^Glued^ staining in approximately 100% of tyrosine hydrogenase (TH)-positive DAergic neurons, while the p135+ staining remained (Fig. 1d, e). Additionally, the levels of p150^Glued^, p135+, and other dynactin subunits were comparable in the olfactory bulb, cerebral cortex, hippocampus, striatum, cerebellum, and brainstem of Ctrl and cKO mice (Fig. 1f, g). Therefore, we deleted p150^Glued^ but kept p135+ expression in midbrain DAergic neurons of cKO mice. Apart from the midbrain DAergic neurons, we examined the Cre-mediated deletion of p150^Glued^ in other types of TH-expressing cells in the central nervous system and peripheral tissues of 1-month-old cKO mice. Interestingly, deletion of p150^Glued^ was observed in approximately 46%, 2%, 99%, and 98% of TH^+^ cells in the *locus coeruleus*, olfactory bulb, superior cervical ganglion, and adrenal medulla, respectively, indicating the differential efficiencies of Cre-mediated recombination in various populations of TH-expressing cells in cKO mice (Supplementary Fig. 1).

In a parallel study, we crossbred *Dctn1*^Loxp/^ mice with the tamoxifen-inducible *Cre/Esr1* transgenic mice^34^ to generate *Dctn1*^+/+^ (WT), *Dctn1*^+/+^;*Cre/Esr1* (Cre), *Dctn1*^Loxp/LoxP^ (Ctrl), and *Dctn1*^LoxP/LoxP^;*Cre/Esr1* [inducible knockout (iKO)] mice. We isolated the midbrain tissues from neonatal iKO pups and littermate controls for primary neuronal cultures. At 1 day in vitro (DIV), we added 1 μM 4-OHT to the medium of primary cultures to induce Cre recombinase activity within cells. At 14 DIV, western blotting and immunocytochemistry confirmed the inducible deletion (~97%) of p150^Glued^ and the compensatory increase (around 92%) of p135+ in the iKO midbrain cell culture, including the DAergic neurons (Supplementary Fig. 2). The *Dctn1*^Loxp/LoxP^;*Cre/Esr1* iKO mice allow for in-depth cell biology and biochemical studies on the functional significance of p150^Glued^ and its MTBDs.

### P150^Glued^ cKO mice exhibit aggravated deterioration of motor coordination during aging

**Fig. 2:**
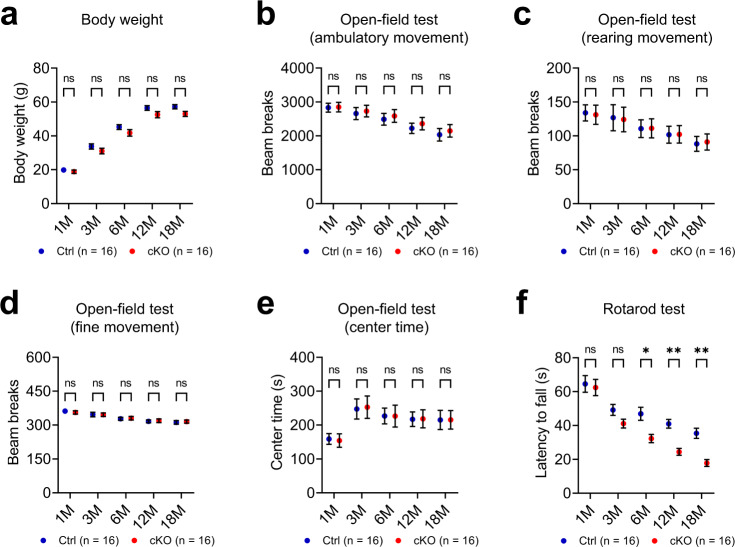
Aggravated deterioration of motor coordination in cKO mice during aging. Cohorts of male Ctrl and cKO mice (*n* = 16 animals per genotype) were repeatedly assessed for body weight and behavioral performance at 1, 3, 6, 12, and 18 months of age. **a** The body weight of Ctrl and cKO mice. **b**–**e** The ambulatory movement (**b**), rearing movement (**c**), fine movement (**d**), and center time (**e**) of Ctrl and cKO mice in the open-field test. **f** The latency to fall of Ctrl and cKO mice in the rotarod test. Data were presented as mean ± SEM. Two-way ANOVA with Sidak’s multiple comparisons test was used for statistical analysis. In the rotarod test (**f**), ^*^p = 0.0104 (6 M), ^**^p = 0.0027 (12 M), ^***^p = 0.0012 (18 M).

P150^Glued^ cKO mice developed normally with no gross physical or behavioral abnormalities. Although weight loss is a typical clinical manifestation of PS patients^9–13^, the cKO mice weighed similarly to the Ctrl mice at 1, 3, 6, 12, and 18 months of age (Fig. 2a). In the open-field test, the cKO mice displayed similar locomotor activities (ambulatory, rearing, and fine movement) and time spent in the center of the arena with the Ctrl mice at 1, 3, 6, 12, and 18 months of age (Fig. 2b–e). In the rotarod test, the cKO mice performed as well as the Ctrl mice at 1 and 3 months, but exhibited a more profound deterioration of rotarod performance than the Ctrl mice starting at 6 months of age (Fig. 2f). Considering the crucial role of dopamine in motor control, the accelerated impairment of motor coordination observed in the cKO mice could result from progressive dysfunction/degeneration of midbrain DAergic neurons during aging.

### P150^Glued^ cKO mice display progressive degeneration of DAergic neurons in the midbrain

**Fig. 3:**
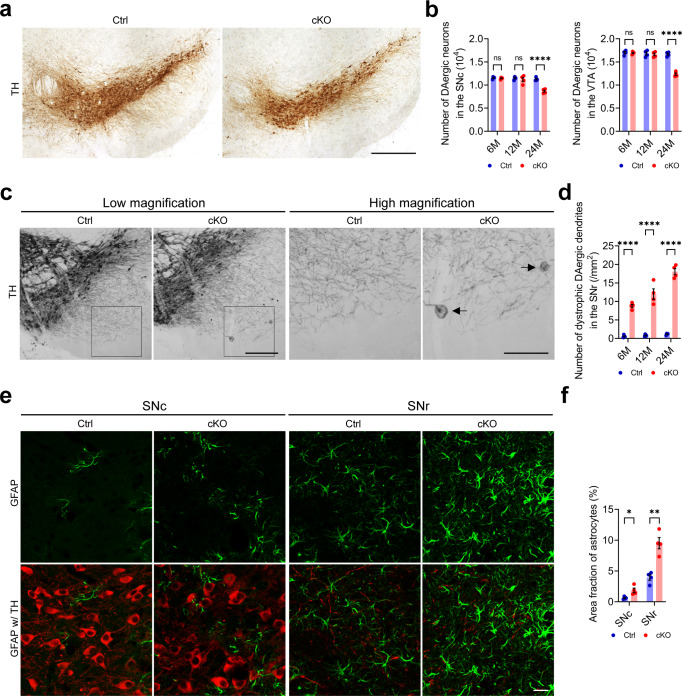
Progressive degeneration of DAergic neurons in the midbrain of cKO mice. **a**, **b** Immunohistochemical images show TH staining in the midbrain of 24-month-old Ctrl and cKO mice. Scale bar: 400 μm. Unbiased stereological estimation of the number of TH-positive DAergic neurons in the SNc and VTA of 6-, 12-, and 24-month-old Ctrl and cKO mice (*n* = 4 animals per genotype per time point). Data were presented as mean ± SEM. Two-way ANOVA, ^****^p < 0.0001. **c**, **d** Representative images show TH staining in the midbrain of 6-month-old Ctrl and cKO mice. Arrows point to the dystrophic DAergic dendrites in the SNr of cKO mice. Dystrophic DAergic dendrites were defined as TH-positive neuritic varicosity ≥25 μm^2^. Scale bar: 200 μm (low magnification), 100 μm (high magnification). The bar graph quantifies the density of dystrophic DAergic dendrites in the SNr of 6-, 12-, and 24-month-old Ctrl and cKO mice (at each time point, *n* = 4 animals per genotype and 5 sections per animal). Data were presented as mean ± SEM. Two-way ANOVA, ^****^p < 0.0001. **e**, **f** Immunofluorescent images show the staining of GFAP (green) and TH (red) in the midbrain of 24-month-old Ctrl and cKO mice. Scale bar: 20 μm. The area fraction of GFAP-positive astrocytes was quantified as mean ± SEM (*n* = 4 animals per genotype and 5 sections per animal). Unpaired *t* test, ^*^p = 0.0178, ^**^p = 0.0016.

Since the loss of midbrain DAergic neurons is the cardinal neuropathological feature of PS^10–13^, we used unbiased stereology to count the TH-positive neurons in the *substantia nigra pars compacta* (SNc) and the ventral tegmental area (VTA) of Ctrl and cKO mice. Compared with the age-matched controls, the cKO mice had similar numbers of midbrain DAergic neurons at 6 and 12 months of age, but significantly fewer DAergic neurons at 24 months of age, with approximately 23 and 26% neuronal loss in the SNc and the VTA, respectively (Fig. 3a, b). Therefore, genetic deletion of p150^Glued^ in midbrain DAergic neurons contribute to late-onset loss of DAergic neurons.

The dendrites of DAergic neurons located in the ventral SNc often protrude perpendicularly into the underneath *substantia nigra par reticulata* (SNr) region and form synaptic connections with the incoming axon fibers^35^. Interestingly, compared with the age-matched controls, the cKO mice displayed substantial and progressive dystrophy of DAergic dendrites (≥25 μm^2^) in the SNr starting at 6 months of age (Fig. 3c, d). Thus, genetic deletion of p150^Glued^ in midbrain DAergic neurons leads to early-onset dystrophy of DAergic dendrites.

In addition to the cell loss and dendritic dystrophy of DAergic neurons, increased astrogliosis was detected in the SNc and the SNr of 24-month-old cKO mice (Fig. 3e, f). As the neuronal loss, the sphere-like neurite swelling, and the gliosis were also observed in the SN of PS patients^9,36,37^, the cKO mice with deletion of p150^Glued^ in the midbrain DAergic neurons recapitulate some key pathological features of PS.

### Abnormal accumulation of α-synuclein but not TDP-43 in the midbrain DAergic neurons of aged p150^Glued^ cKO mice

**Fig. 4:**
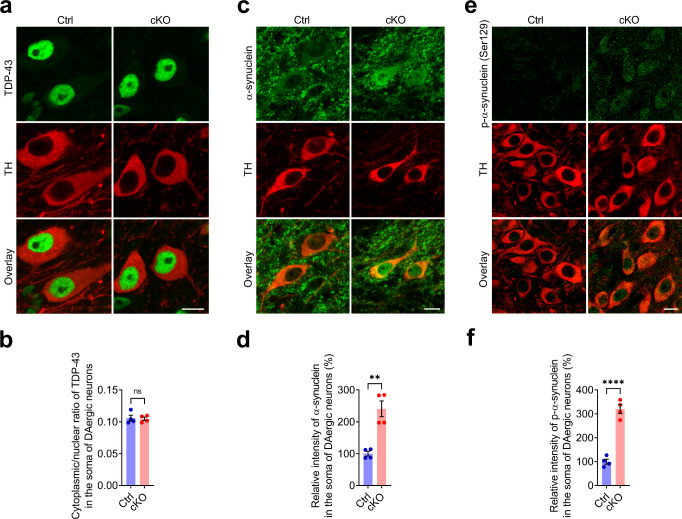
Abnormal accumulation of α-synuclein but not TDP-43 in the midbrain DAergic neurons of cKO mice. **a**, **b** Immunofluorescent images show the staining of TDP-43 (green) and TH (red) in the midbrain of 24-month-old Ctrl and cKO mice. Scale bar: 10 μm. The cytoplasmic/nuclear ratio of TDP-43 in the soma of DAergic neurons was quantified as mean ± SEM (*n* = 4 animals per genotype and ≥30 neurons per animal). Unpaired *t* test, ns (*p* = 0.8547). **c**, **d** Immunofluorescent images show the staining of α-synuclein (green) and TH (red) in the midbrain of 24-month-old Ctrl and cKO mice. Note the increase of cytoplasmic and nuclear α-synuclein in the midbrain DAergic neurons of cKO mice. Scale bar: 10 μm. The staining intensity of α-synuclein in the soma of DAergic neurons was quantified as mean ± SEM (*n* = 4 animals per genotype and ≥30 neurons per animal). Unpaired *t* test, ^**^p = 0.0016. **e**, **f** Immunofluorescent images show the staining of p-α-synuclein (Ser129) (green) and TH (red) in the midbrain of 24-month-old Ctrl and cKO mice. Note the increase of somatic p-α-synuclein (Ser129) in the midbrain DAergic neurons of cKO mice. Scale bar: 10 μm. The staining intensity of p-α-synuclein in the soma of DAergic neurons was quantified as mean ± SEM (*n* = 4 animals per genotype and ≥30 neurons per animal). Unpaired *t* test, ^****^p < 0.0001.

As abnormal TAR DNA-binding protein 43 (TDP-43)-positive cytoplasmic inclusions were often identified in the SN and other brain regions of PS patients^9,36,37^, we examined the subcellular location of TDP-43 in the midbrain DAergic neurons of 24-month-old Ctrl and cKO mice. However, we did not detect any apparent accumulation of TDP-43 in the cytosol of either Ctrl or cKO DAergic neurons (Fig. 4a, b). By contrast, although very sparse Lewy bodies composed of α-synuclein aggregates were identified in the brain of PS patients^10^, we observed a substantial increase (approximately 140%) of cytoplasmic and nuclear accumulation of α-synuclein in the midbrain DAergic neurons of 24-month-old cKO mice compared with the Ctrl mice (Fig. 4c, d). While α-synuclein is typically enriched in the axon terminals, the abnormal accumulation of α-synuclein in cytosol and nucleus is implicated in neurodegeneration^38,39^. It has been reported that α-synuclein in synucleinopathy lesions is extensively phosphorylated at Ser129^40^. In line with this finding, immunostaining revealed a significant increase (~220%) of p-α-synuclein (Ser129) immunoreactivity in the soma of midbrain DAergic neurons of 24-month-old cKO mice compared with the Ctrl mice (Fig. 4e, f). Emerging evidence suggests that α-synuclein pathology can spread intercellularly and between interconnected brain areas^41^. However, we detected no apparent accumulation of α-synuclein in the soma of TH-negative neurons in the SNc, SNr, dorsal striatum, hippocampal CA1 area, and prefrontal cortex of 24-month-old cKO mice (Supplementary Fig. 3). This indicates that α-synuclein pathology in the midbrain DAergic neurons of aged cKO mice rarely spreads to other neurons in the neighboring and interconnected brain areas.

### P150^Glued^ cKO mice show progressive degeneration of DAergic axon terminals in the striatum

**Fig. 5:**
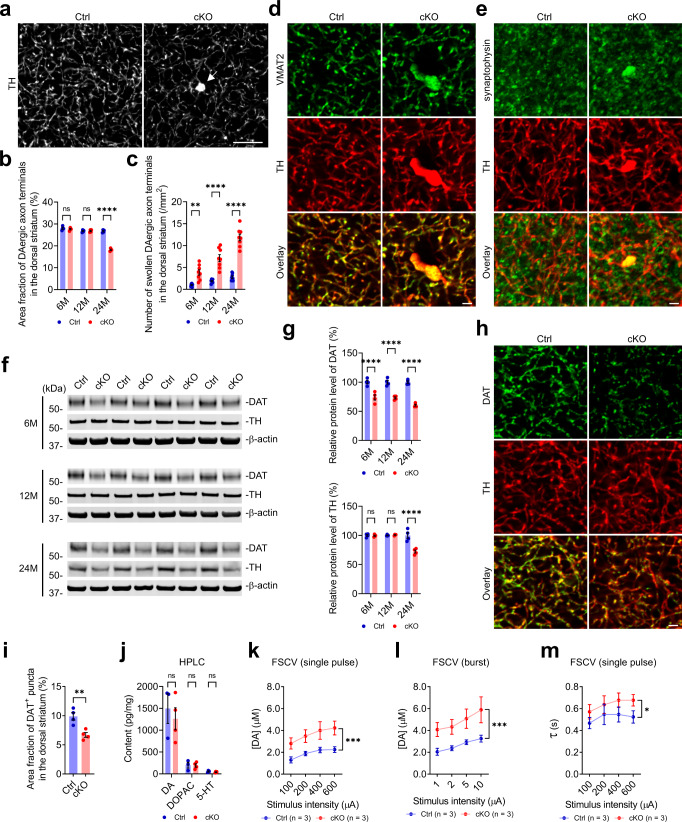
Progressive degeneration of DAergic axon terminals in the striatum of cKO mice. **a**–**c** Representative images show TH staining in the dorsal striatum of 24-month-old Ctrl and cKO mice. The arrow points to the swollen DAergic axon terminal (defined as TH-positive neuritic varicosity ≥3 μm^2^) of cKO mice. Scale bar: 10 μm. The area fraction of DAergic axon terminals (**b**) and density of swollen DAergic axonal terminals (**c**) in the dorsal striatum of 6-, 12-, and 24-month-old Ctrl and cKO mice were quantified as mean ± SEM (at each time point, *n* ≥ 4 animals per genotype and ≥5 sections per animal). Two-way ANOVA, ^**^p = 0.0015, ^****^p < 0.0001. **d**, **e** Immunofluorescent images show the co-staining of VMAT2 (**d**, green) or synaptophysin (**e**, green) with TH (red) in the dorsal striatum of 6-month-old Ctrl and cKO mice. Note the accumulation of VMAT2-positive (**d**) and synaptophysin-positive (**e**) vesicles within the swollen DAergic axon terminal. Scale bar: 2 μm. **f**, **g** Western blots show the expression of DAT and TH in the striatum of 6-, 12-, and 24-month-old Ctrl and cKO mice. The protein level was quantified as mean ± SEM (at each time point, *n* = 4 animals per genotype). Two-way ANOVA, ^****^p < 0.0001. **h**, **i** Immunofluorescent images show the staining of DAT (green) and TH (red) in the dorsal striatum of 6-month-old Ctrl and cKO mice. Scale bar: 2 μm. The area fraction of DAT-positive puncta was quantified as mean ± SEM (*n* = 4 animals per genotype and 5 sections per animal). Unpaired *t* test, ^**^p = 0.0060. **j** HPLC measures the content of DA, DOPAC, and 5-HT in the dorsal striatum of 12-month-old Ctrl (*n* = 3) and cKO (*n* = 4) mice. Data were presented as mean ± SEM. Unpaired *t* test, ns (*p* ≥ 0.05). **k**–**m** FSCV quantifies the kinetics of evoked DA release in the dorsal striatum of 12-month-old Ctrl and cKO mice (*n* = 3 animals per genotype and 3 sections per animal). Data were presented as mean ± SEM. For the peak evoked DA release following single-pulse electrical stimulation of different stimulus intensities (**k**), Two-way ANOVA genotype factor: *F*_(1, 16)_ = 25.11, ^***^p = 0.0001. For the peak evoked DA release in response to burst electrical stimulation (50 Hz, 5 pulses) of different stimulus intensities (**l**), Two-way ANOVA genotype factor: *F*_(1, 16)_ = 23.09, ^***^p = 0.0002. For the time constant of slope decay (*τ*) following single-pulse electrical stimulation of different stimulus intensities (**m**), Two-way ANOVA genotype factor: *F*_(1, 16)_ = 6.035, ^*^p = 0.0258.

As axon dystrophy was reported in the brains of PS patients^9,36,37^, we examined the density and morphology of DAergic axon terminals in the dorsal striatum of Ctrl and cKO mice. Compared with the age-matched controls, the cKO mice had similar densities of striatal DAergic axon terminals at 6 and 12 months of age, but significantly sparser striatal DAergic axon terminals at 24 months of age, with approximately 32% of axonal loss (Fig. 5a, b). This finding agrees with the late-onset loss of midbrain DAergic neurons in the cKO mice (Fig. 3a, b). TH staining also revealed gradually increasing numbers of abnormally swollen DAergic axon terminals (≥3 μm^2^) in the dorsal striatum of 6-, 12-, and 24-month-old cKO mice compared with the age-matched controls (Fig. 5a, c). In addition, the swollen axon terminals in cKO mice were packed with VMAT2- and synaptophysin-positive vesicles (Fig. 5d, e). These data show that genetic deletion of p150^Glued^ in midbrain DAergic neurons induces early-onset swelling and late-onset loss of DAergic axon terminals in the striatum.

As neuroimaging has shown marked loss of dopamine transporter (DAT) binding in the striatum of PS patients^14–16^, we examined the protein level of DAT, which is crucial for dopamine uptake, in the striatal homogenates of 6-, 12-, and 24-month-old Ctrl and cKO mice. Western blotting revealed a substantial decrease of DAT in the striatum of cKO mice compared with age-matched controls, with ~26%, 27%, and 39% of DAT loss at 6, 12, and 24 months of age, respectively (Fig. 5f, g). Immunostaining further confirmed a marked reduction (around 32%) of DAT-positive puncta in the dorsal striatum of 6-month-old cKO mice compared with Ctrl mice (Fig. 5h, i). These results demonstrate that genetic deletion of p150^Glued^ in midbrain DAergic neurons contribute to the early-onset reduction of DAT in the striatum.

In parallel, we examined the protein level of TH, the rate-limiting enzyme in DA synthesis, in the striatal homogenates of 6-, 12-, and 24-month-old Ctrl and cKO mice. Compared with the age-matched controls, the cKO mice showed no marked change of TH at 6 and 12 months of age but a substantial reduction (about 28%) of TH at 24 months of age (Fig. 5f, g), consistent with the late-onset loss of striatal DAergic axon terminals in cKO mice (Fig. 5a, b). In correlation with no apparent loss of DAergic axon terminals or TH expression in the cKO mice at 12 months of age (Fig. 5b, f, g), HPLC revealed similar content of DA, dopamine metabolite 3,4-dihydroxyphenylacetic acid (DOPAC), or 5-hydroxytryptamine (5-HT) in the dorsal striatum of 12-month-old cKO mice compared with controls (Fig. 5j).

Considering the early-onset morphological and biochemical changes of DAergic axon terminals in the cKO mice (Fig. 5c–i), we further evaluated the kinetics of DA release and uptake in the striatal slices prepared from 12-month-old Ctrl and cKO mice using fast-scan cyclic voltammetry (FSCV). In response to either single- or burst-pulse stimulation, the peak evoked DA release was substantially higher from the DAergic axon terminals of the cKO mice than the controls (Fig. 5k, l). Additionally, the time constant of slope decay (τ), which measures the kinetics of dopamine uptake, was also significantly increased in the DAergic axon terminals of the cKO mice compared with the controls (Fig. 5m). Taken together, these data show that genetic deletion of p150^Glued^ in midbrain DAergic neurons leads to early-onset swelling of DAergic axon terminals accumulated with DA-containing vesicles, reduction of DAT, and the resultant dysregulation of DA transmission in the dorsal striatum, which may contribute to the early-onset impairment of motor coordination in the cKO mice.

### P150^Glued^ deficiency induces reorganization of ER structure and increases ER tubule-shaping protein reticulon 3 (RTN3)

**Fig. 6:**
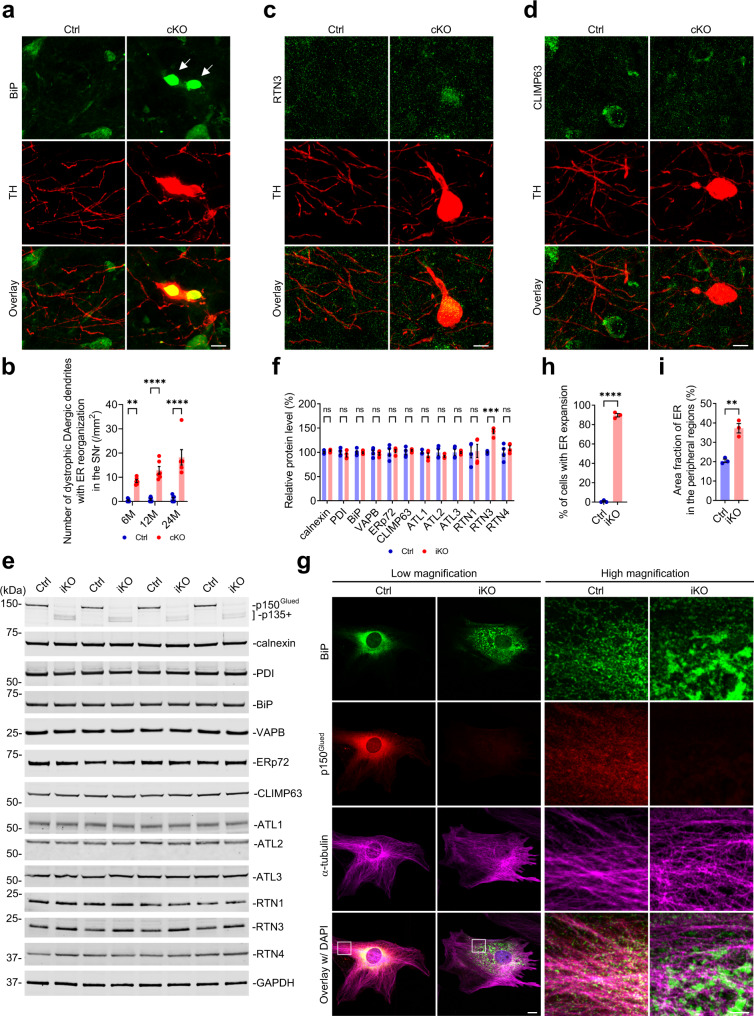
P150^Glued^ deficiency induces reorganization of ER structure and increases ER tubule-shaping protein RTN3. **a**, **b** Immunofluorescent images show the staining of BiP (the ER marker, green) and TH (red) in the SNr of 6-month-old Ctrl and cKO mice. Arrows point to the abnormal reorganization of ER within the dystrophic DAergic dendrite of cKO mice. Scale bar: 10 μm. The Bar graph quantifies the density of dystrophic DAergic dendrites with ER reorganization in the SNr of 6-, 12-, and 24-month-old Ctrl and cKO mice (at each time point, *n* = 6 animals per genotype and 4 sections per animal). Data were presented as mean ± SEM. Two-way ANOVA, ^**^p = 0.0020, ^****^p < 0.0001. **c**, **d** Immunofluorescent images show the co-staining of ER tubule-shaping protein RTN3 (**c**, green) or ER sheet-shaping protein CLIMP63 (**d**, green) with TH (red) in the SNr of 6-month-old Ctrl and cKO mice. Note the positive immunoreactivity of RTN3 (**c**) and the negative immunoreactivity of CLIMP63 (**d**) in the dystrophic DAergic dendrite of cKO mice. Scale bar: 10 μm. **e**, **f** Western blots, show the expression of ER-resident proteins (calnexin, PDI, BiP, VAPB, and ERp72) and ER-shaping proteins (CLIMP63, ATL1, ATL2, ATL3, RTN1, RTN3, and RTN4) in the Ctrl and iKO fibroblasts at 14 DIV. The protein level was quantified as mean ± SEM (*n* = 4 independent cultures). Unpaired *t* test, ^***^p = 0.0001. **g**–**i** Immunofluorescent images show the staining of BiP (green), p150^Glued^ (red), α-tubulin (magenta), and DAPI (blue) in the Ctrl and iKO fibroblasts at 14 DIV. Note the obvious expansion of perinuclear ER and the abnormal reorganization of peripheral ER in the iKO fibroblast. Scale bar: low magnification, 20 μm; high magnification, 5 μm. The percentage of cells with ER expansion (**h**) and the area fraction of ER in the peripheral regions were quantified as mean ± SEM (*n* = 3 independent cultures and ≥60 cells per genotype). Unpaired *t* test, ^**^p = 0.0028^, ****^p < 0.0001.

To investigate the composition of dystrophic DAergic dendrites in the SNr of cKO mice, we co-stained the midbrain sections from 6-month-old Ctrl and cKO mice with antibodies against TH and various intracellular organelle markers. The occupancy of ER (visualized by BiP, calnexin, or PDI staining), Golgi (visualized by RCAS1 staining), endosomes (visualized by EEA1 staining), autophagosomes (visualized by SQST1 staining), and lysosomes (visualized by cathepsin D staining) in the dystrophic DAergic dendrites was ~80%, 0%, 7%, 11%, and 12%, respectively (Fig. 6a and Supplementary Fig. 4a–g). These findings indicate that the reorganized ER is the major organelle within the dystrophic DAergic dendrite of cKO mice. Three-dimensional (3D) reconstruction of the ER and DAergic dendrites in the SNr of 6-month-old Ctrl and cKO mice further demonstrated that the reorganized ER occupied a large portion of the intracellular space of the dystrophic DAergic dendrite in cKO mice (Supplementary Fig. 4h and Supplementary Movie 1 and 2). Moreover, the number of dystrophic DAergic dendrites with ER reorganization in the SNr of cKO mice increased in an age-dependent manner, ~8.42, 12.91, and 18.25 per mm^2^ at 6, 12, and 24 months of age, respectively (Fig. 6a, b).

The ER network consisting of interconnected tubules and sheets extends throughout the neuron and adopts heterogeneous architecture in soma, axon, and dendrites^42,43^. Several classes of proteins are enriched in ER tubules or sheets and participate in the shaping of ER membranes^44–46^. We detected positive immunoreactivity of ER tubule-shaping protein RTN3 in the dystrophic DAergic dendrites of 6-month-old cKO mice (Fig. 6c), but negative immunoreactivity of ER sheet-shaping protein CLIMP63 (Fig. 6d). These observations suggest the participation of RTN3 in the ER reorganization within the dystrophic DAergic dendrites of cKO mice. Western blotting of Ctrl and iKO fibroblasts revealed that p150^Glued^ deficiency selectively increased (approximately 43%) the protein level of RTN3 without changing the expression of ER-resident proteins (including calnexin, PDI, BiP, VAPB, and ERp72) and other ER-shaping proteins (including CLIMP63, ATL1, ATL2, ATL3, RTN1, RTN4) (Fig. 6e, f). Using immunocytochemistry, we further examined the subcellular distribution pattern and fine structure of ER in the Ctrl and iKO fibroblasts. BiP, α-tubulin, and p150^Glued^ co-staining revealed well-organized ER architecture in the Ctrl fibroblasts, with cohesive ER sheets concentrating in the perinuclear regions and three-way junction of ER tubules distributing in the peripheral areas (Fig. 6g–i). By contrast, over 89% iKO fibroblasts exhibited expansion of perinuclear ER (Fig. 6g–i). Compared with the Ctrl fibroblasts, the iKO fibroblasts exhibited a significantly increased (~82%) area fraction of ER in the peripheral regions, with evident clustering of ER tubules and the existence of ER sheets (Fig. 6g–i). Together, these findings demonstrate that p150^Glued^ deficiency induces ER reorganization and selectively increases ER tubule-shaping protein RTN3 level. Interestingly, RTN3-immunoreactive dystrophic neurites have been found in Alzheimer’s disease brains, and DCTN6 deficiency enhanced RTN3 protein level and the formation of RTN3-immunoreactive dystrophic neurites in the hippocampus of aging mice^47,48^.

### P150^Glued^ deficiency induces defects in the early secretory pathway and impairs the COPII-mediated ER export

**Fig. 7:**
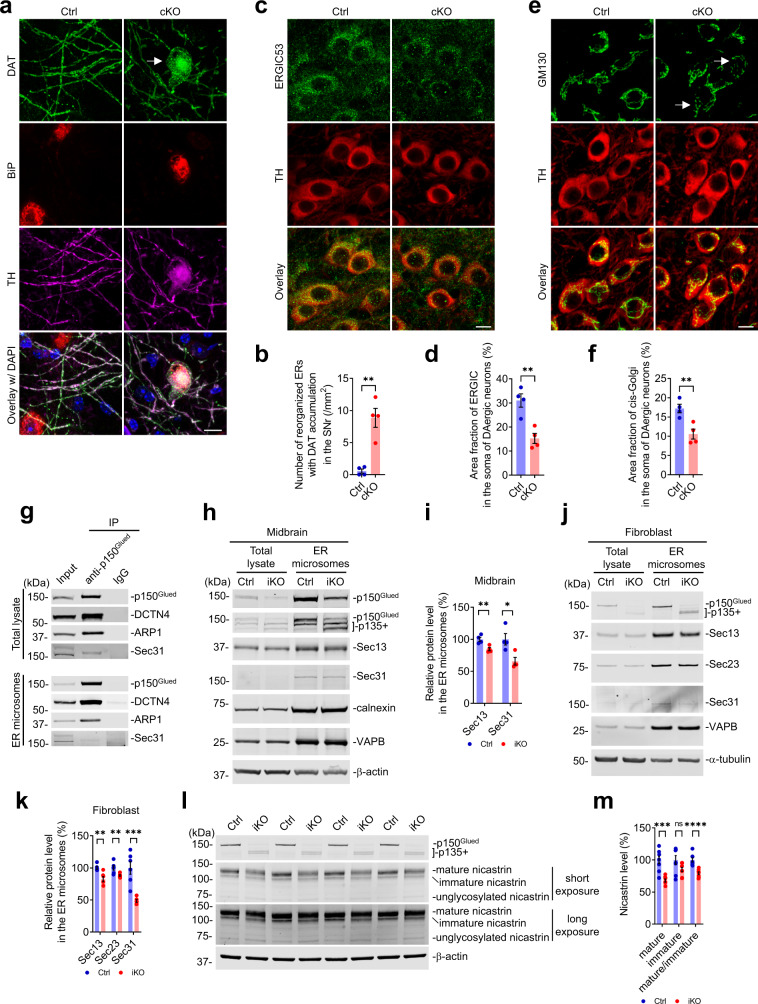
P150^Glued^ deficiency induces defects in the early secretory pathway and impairs the COPII-mediated ER export. **a**, **b** Immunofluorescent images show the staining of DAT (green), BiP (red), TH (magenta), and DAPI (blue) in the SNr of 6-month-old Ctrl and cKO mice. The arrow points to the accumulation of DAT inside the reorganized ER within the dystrophic DAergic dendrite of cKO mice. Scale bar: 10 μm. The density of reorganized ERs with DAT accumulation was quantified as mean ± SEM (*n* = 4 animals per genotype and 4 sections per animal). Unpaired *t* test, ^**^p = 0.0016. **c**–**f** Immunofluorescent images show the co-staining of ERGIC53 (**c**, the ERGIC marker, green) or GM130 (**e**, the cis-Golgi marker, green) with TH (red) in the midbrain of 6-month-old Ctrl and cKO mice. Scale bar: 10 μm. The area fractions of ERGIC (**d**) and cis-Golgi (**f**) in the soma of DAergic neurons were quantified as mean ± SEM (*n* = 4 animals per genotype and ≥30 neurons per animal). Unpaired *t* test, ^**^p = 0.0039 (**d**), ^**^p = 0.0079 (**f**). **g** Co-IP reveals interaction between p150^Glued^ and COPII component Sec31 in the mouse brains’ total lysate and ER microsomes. **h**, **i** Western blots show the expression of COPII components (Sec13 and Sec31) in the midbrains’ total lysate and ER microsomes from 4-month-old Ctrl and iKO mice. The Ctrl and iKO mice were intraperitoneally injected with tamoxifen at 3 months of age to induce Cre recombinase activity and the resultant deletion of p150^Glued^. Calnexin and VAPB were used as markers of ER microsomes. The protein level in the ER microsomes was quantified as mean ± SEM (*n* = 4 independent experiments). Unpaired *t* test, ^*^p = 0.0187, ^**^p = 0.0090. **j**, **k** Western blots show the expression of COPII components (Sec13, Sec23, and Sec31) in the total lysate and ER microsomes of Ctrl and iKO fibroblasts at 14 DIV. The protein level in the ER microsomes was quantified as mean ± SEM (*n* = 6 independent cultures). Unpaired *t* test, ^**^p = 0.0032 (Sec13), ^**^p = 0.0097 (Sec23), ^***^p = 0.0005. **l**, **m** Western blots show the expression of mature, immature, and unglycosylated nicastrin in the Ctrl and iKO fibroblasts at 14 DIV. The protein level and mature/immature ratio of nicastrin were quantified as mean ± SEM (*n* = 8 independent cultures). Unpaired *t* test, ^***^p = 0.0002, ^****^p < 0.0001.

The early secretory pathway, consisting of ER, ER*-*Golgi intermediate compartment (ERGIC), and Golgi apparatus, ensures the proper supply of secretory and membrane materials for neurons, playing an essential role in neuronal survival and function^49,50^. Immunohistochemistry revealed a severe accumulation of DAT inside the reorganized ER within the dystrophic DAergic dendrite of 6-month-old cKO mice, indicating the potential dysfunction of ER export (Fig. 7a, b). The findings that DAT protein was trapped in the reorganized ER of p150^Glued^-deficient DAergic neurons may explain the reduction of DAT protein in the axon terminals of cKO mice (Fig. 5f–i). Furthermore, we found a marked reduction (approximately 51%) of the area fraction of ERGIC (visualized by ERGIC53 staining) in the soma of midbrain DAergic neurons from 6-month-old cKO mice compared with the controls (Fig. 7c, d). In addition, the area fraction of cis-Golgi (visualized by GM130 staining) was also significantly decreased (~39%) in the soma of p150^Glued^-deficient DAergic neurons, while Golgi fragmentation was apparent in individual p150^Glued^-deficient DAergic neurons of the cKO mice at 6 months of age (Fig. 7e, f). These data demonstrate that p150^Glued^ deficiency induces defects in the early secretory pathway of midbrain DAergic neurons.

Previous studies demonstrate that p150^Glued^ stabilizes the coat protein complex II (COPII) at ER exit site (ERES) through interaction with COPII component Sec23^51,52^. Consistent with the early findings, co-immunoprecipitation (co-IP) revealed endogenous protein-protein interaction between p150^Glued^ and COPII component Sec31 in mouse brain’s total lysate and ER microsomes (Fig. 7g). Moreover, we found that COPII components Sec13 and Sec31 substantially decreased in the midbrain’s ER microsomes of 4-month-old iKO mice compared with the Ctrl mice (Fig. 7h, i). The reduction of Sec13, Sec23, and Sec31 was also observed in the ER microsomes isolated from the cultured iKO fibroblasts compared to the Ctrl cells (Fig. 7j, k). The concentration of COPII at ERES is critical for ER export of secretory and membrane proteins, such as type-I integral membrane protein nicastrin, which is partially glycosylated in ER as the immature form and then exported to Golgi apparatus for fully glycosylation and maturation^53^. The ratio of mature versus immature nicastrin can be used to estimate the efficiency of ER export in cells^53,54^. Accordingly, compared with Ctrl cells, the iKO fibroblasts displayed a substantial decrease in the level of mature nicastrin and the ratio of mature/immature nicastrin (Fig. 7l, m), implying dysfunction of ER export in the p150^Glued^-deficient cells. Therefore, our studies further support that p150^Glued^ deficiency destabilizes the COPII complex at ERES and compromises the COPII-mediated ER export.

### P150^Glued^ deficiency activates unfolded protein response and exacerbates ER stress-induced DAergic neuron death

**Fig. 8:**
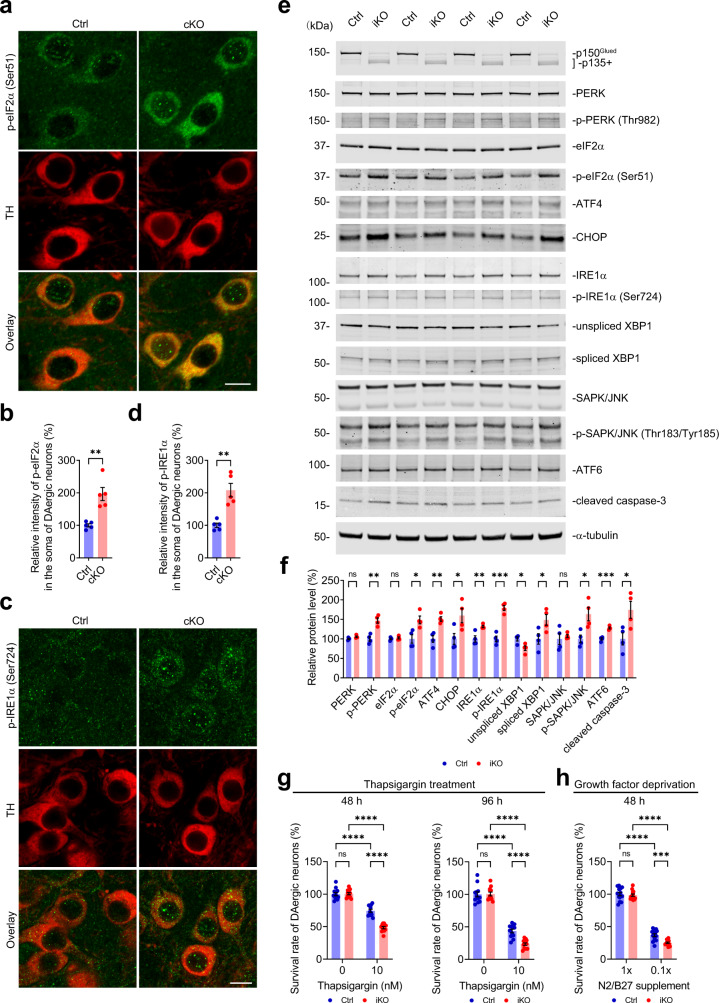
P150^Glued^ deficiency activates unfolded protein response and exacerbates ER stress-induced cell death of DAergic neurons. **a**–**d** Immunofluorescent images show the co-staining of p-eIF2α (Ser51) (**a**, green) or p-IRE1α (Ser724) (**c**, green) with TH (red) in the midbrain of 18-month-old Ctrl and cKO mice. Scale bar: 10 μm. The staining intensity of p-eIF2α (**b**) or p-IRE1α (**d**) in the soma of DAergic neurons was quantified as mean ± SEM (*n* = 5 animals per genotype and ≥20 neurons per animal). Unpaired *t* test, ^**^p = 0.0016 (**b**), ^**^p = 0.0011 (**d**). **e**, **f** Western blots show the expression of UPR (PERK-eIF2α-ATF4-CHOP, IRE1-XBP1-SAPK/JNK, and ATF6) and cell death (cleaved caspase-3) regulatory proteins in the Ctrl and iKO fibroblasts at 14 DIV. The protein level was quantified as mean ± SEM (*n* = 4 independent cultures). Unpaired *t* test, ^**^p = 0.0017 [p-PERK (Thr982)], ^*^p = 0.0159 [p-eIF2α (Ser51)], ^**^p = 0.0028 (ATF4), ^*^p = 0.0405 (CHOP), ^**^p = 0.0096 (IRE1α), ^***^p = 0.0002 [p-IRE1α (Ser724)], ^*^p = 0.0237 (unspliced XBP1), ^*^p = 0.0451 (spliced XBP1), ^*^p = 0.0190 [p-SAPK/JNK (Thr183/Tyr185)], ^***^p = 0.0003 (ATF6), ^*^p = 0.0317 (cleaved caspase-3). **g** Bar graphs show the survival rates of Ctrl and iKO midbrain DAergic neurons (14 DIV) treated with 0 or 10 nM thapsigargin for 48 and 96 h (at each time point, *n* = 12 coverslips per genotype per condition). 10 nM thapsigargin was used to induce ER stress. Data were presented as mean ± SEM. One-way ANOVA, ^****^p < 0.0001. h The bar graph shows the survival rates of Ctrl and iKO midbrain DAergic neurons (14 DIV) cultured in the presence of 1× or 0.1× N2/B27 supplement for 48 h (*n* = 14 coverslips per genotype per condition). The content of N2/B27 supplement in the culture medium was lowed from 1× to 0.1× to induce growth factor deprivation. Data were presented as mean ± SEM. One-way ANOVA, ^***^p = 0.0008, ^****^p < 0.0001.

Since the disruption of ER structural or functional homeostasis triggers ER stress and unfolded protein response (UPR)^55,56^, we investigated the effects of p150^Glued^ deficiency on a series of proteins implicated in the UPR pathway. Using immunohistochemistry, we detected a marked increase (approximately 96%) of phosphorylated eIF2α (Ser51) [p-eIF2α (Ser51)] in the midbrain DAergic neurons of 18-month-old cKO mice compared with Ctrl mice (Fig. 8a, b). Additionally, a significant upregulation (approximately 108%) of phosphorylated IRE1α (Ser724) [p-IRE1α (Ser724)] was observed in the midbrain DAergic neurons of 18-month-old cKO mice compared with Ctrl mice (Fig. 8c, d). By western blotting, we found a substantial increase of phosphorylated PERK (Thr982) [p-PERK (Thr982)], p-eIF2α (Ser51), ATF4, and CHOP in the iKO fibroblasts compared to the Ctrl cells (Fig. 8e, f), indicating that p150^Glued^ deficiency leads to the activation of the PERK-eIF2α-ATF4-CHOP branch of UPR pathway. We also found a significant upregulation of IRE1α, p-IRE1α (Ser724), spliced XBP1, and phosphorylated SPK/JNK (Thr183/Tyr185) [p-SPK/JNK (Thr183/Tyr185)], as well as a marked decrease of unspliced XBP1, in the iKO fibroblasts (Fig. 8e, f), demonstrating the activation of the IRE1-XBP1-SAPK/JNK branch of UPR pathway due to p150^Glued^ deficiency. Additionally, the increased expression of ATF6 revealed the activation of the ATF6 branch of UPR pathway in the iKO fibroblasts (Fig. 8e, f). Moreover, the elevated level of cleaved caspase-3 indicates the activation of the cell death pathway in the iKO fibroblasts (Fig. 8e, f).

Despite the beneficial role of UPR in sensing and mitigating ER stress, the sustained UPR activation under irremediable ER stress can result in cell death, contributing to the pathogenesis of a wide range of human diseases, including neurodegeneration^55,56^. To determine whether p150^Glued^ deficiency affects the susceptibility of midbrain DAergic neurons to ER stress-induced cell death, we treated Ctrl and iKO midbrain neuron cultures (14 DIV) with 0 or 10 nM thapsigargin, an inhibitor of ER Ca^2+^-ATPase. After treating with 10 nM thapsigargin for 48 h, the survival rates of Ctrl and iKO midbrain DAergic neurons were ~74 and 48%, respectively (Fig. 8g). After treating with 10 nM thapsigargin for 96 h, the survival rates of Ctrl and iKO midbrain DAergic neurons further fell to 44 and 23%, respectively (Fig. 8g). At both time points, thapsigargin-induced cell death was exacerbated significantly in the iKO midbrain DAergic neurons, as compared with the Ctrl midbrain DAergic neurons (Fig. 8g). Similarly, the iKO midbrain DAergic neurons were more susceptible to cell death induced by growth factor deprivation (Fig. 8h), another type of cellular stress previously shown to induce apoptosis^57^. To investigate whether inhibition of UPR signaling has protective activity against ER stress-induced cell death of midbrain DAergic neurons, we applied GSK2606414 (an inhibitor of PERK) and KIRA8 (an inhibitor of IRE1α). Co-application of 100 nM GSK2606414 or 100 nM KIRA8 with 10 nM thapsigargin for 48 h significantly mitigated thapsigargin-induced cell death of both the Ctrl and iKO midbrain DAergic neurons (Supplementary Fig. 5). Collectively, our data demonstrate that p150^Glued^ deficiency activates unfolded protein response, making the DAergic neurons more susceptible to ER stress-induced cell death.

## Discussion

In the present study, we generated *Dctn1*^LoxP/LoxP^;*Th-Cre* cKO mice by abolishing p150^Glued^ expression but keeping p135+ expression in midbrain DAergice neurons. We found the accelerated deterioration of motor coordination and progressive degeneration of nigrostriatal DAergic pathway in the cKO mice. Compared with the age-matched controls, the cKO mice exhibited late-onset loss of DAergic neurons and axon terminals, as well as somatic accumulation of α-synuclein. Prior to the neuronal and axonal loss, the cKO mice exhibited early-onset dystrophy of DAergic dendrites, swelling of DAergic axon terminals with the accumulation of VMAT2-and synaptophysin-positive vesicles, reduction of striatal DAT, and the resultant dysregulation of striatal DA transmission. At the subcellular and molecular level, we revealed that p150^Glued^ deficiency induced ER reorganization within the dystrophic DAergic dendrites and upregulated ER tubule-shaping protein RTN3. Meanwhile, p150^Glued^ deficiency led to multiple defects in the early secretory pathway of DAergic neurons, including DAT accumulation inside the reorganized ER and diminished area fractions of ERGIC and cis-Golgi. Moreover, we demonstrated that p150^Glued^ interacted with COPII component Sec31, and p150^Glued^ deficiency impaired the ER export mediated by COPII. Finally, we provided evidence that p150^Glued^ deficiency activated UPR in DAergic neurons and made DAergic neurons more susceptible to ER stress-induced cell death, suggesting that ER structural and functional abnormalities may contribute to the degeneration of midbrain DAergic neurons in PS.

The identification of p150^Glued^ mutations as the genetic cause of PS promotes the studies on the roles of p150^Glued^ in the survival and function of midbrain DAergic neurons^9–13^. Recently, G71A p150^Glued^ KI mice have been generated^31^. Homozygous G71A p150^Glued^ KI mice show the same early embryonic lethality as the homozygous p150^Glued^ KO mice^27,31^, indicating that the PS-linked missense mutations compromise the function of p150^Glued^. Heterozygous G71A p150^Glued^ KI mice are viable and fertile, displaying behavioral defects in tail-suspension and beam-walking tests and a decrease in the TH immunoreactivity of DAergic neurons^31^. Additionally, using a tetracycline-controlled transcriptional regulation system, we generated transgenic mice with selective overexpression of G71R p150^Glued^ in the midbrain DAergic neurons^32^. The G71R p150^Glued^ transgenic mice exhibited early-onset dysregulation of striatal DA transmission and the resultant motor abnormalities, as well as late-onset loss of DAergic neurons and axon terminals^32^. In line with these earlier findings, our current study further highlighted the essential role of p150^Glued^ and its MTBDs in the survival and function of midbrain DAergic neurons. We observed early-onset impairment of motor coordination, dystrophy of DAergic dendrites, swelling of DAergic axon terminals, and dysregulated DA transmission in the cKO mice. We also found late-onset loss of DAergic neurons and axons in the cKO mice. These observations demonstrate that the loss-of-function of MTBDs in p150^Glued^ is sufficient to lead to dysfunction and degeneration of midbrain DAergic neurons.

The MTBDs in p150^Glued^ are required to initiate retrograde axonal transport and to maintain axon terminals’ integrity^23–25^. Accordingly, axonal pathologies of spinal motor neurons, such as axonal swellings, axon terminal degeneration, and accumulation of synaptic vesicles in neuromuscular junctions, have been observed in the KI and transgenic mice expressing MND-related G59S mutant p150^Glued^, as well as the *Dctn1*^LoxP/LoxP^;*Thy1-Cre* mice which lack the MTBDs-containing p150^Glued^ but express p135+ in the forebrain and spinal neurons^22,27–29^. Similarly, substantial loss and abnormal swellings of DAergic axon terminals have been found in transgenic mice with selective overexpression of PS-related G71R mutant p150^Glued^ in the midbrain DAergic neurons^32^. In line with these findings, our current study revealed early-onset swelling of DAergic axon terminals with the accumulation of DA-containing vesicles, reduction of DAT, and the resultant dysregulation of DA transmission in the dorsal striatum of cKO mice. In addition, we detected late-onset loss of DAergic axon terminals in the dorsal striatum of cKO mice. Interestingly, the cKO mice displayed early-onset dystrophy of DAergic dendrites in the SNr, indicating an essential role of p150^Glued^ in maintaining the structural integrity of both axons and dendrites of DAergic neurons. The substantial pathological changes of DAergic axons and dendrites might compromise the function of DAergic circuitry and contribute to the eventual death of DAergic neurons. Thus, future studies will be needed to unmask the mechanisms underlying the early-onset degeneration of DAergic axons and dendrites in PS.

Since the MND- and PS-linked missense mutations in p150^Glued^ all reside within or close to the CAP-Gly domain and compromise the function of MTBDs^8–13,23–26^, the MTBDs in p150^Glued^ must participate in some distinct cellular processes essential for the survival and function of spinal motor neurons and midbrain DAergic neurons. In a recent study, we genetically deleted p150^Glued^ but kept p135+ expression in the forebrain and spinal neurons and found no widespread neuronal loss but selective degeneration of spinal motor neurons^22^. Further histological and biochemical assays revealed that the lack of MTBDs in p150^Glued^ promoted acetylation of microtubules, impairment of the autophagosome-lysosome pathway, cell surface targeting of ionotropic glutamate receptors, and vulnerability to glutamate-induced excitotoxicity in spinal motor neurons^22^. Here when we genetically deleted p150^Glued^ but kept p135+ in midbrain DAergic neurons, we found that p150^Glued^ deficiency particularly affected the structure and function of ER. The lack of MTBDs in p150^Glued^ led to a substantial reorganization of ER in the dystrophic DAergic dendrites, a preferential increase of ER tubule-shaping protein RTN3, accumulation of DAT within the reorganized ER, decreased ER targeting of COPII, dysfunction of ER export, activation of UPR, and increased susceptibility to ER stress-induced cell death in midbrain DAergic neurons. These findings highlight the important role of p150^Glued^ and its MTBDs in maintaining the structural and functional homeostasis of ER, which is critical for the survival and function of midbrain DAergic neurons. Since the carboxyl terminus of DAT protein also binds to COPII component SEC24 and genetic knockdown of SEC24 impairs the cell surface targeting of DAT^58,59^, the impaired ER export likely also contributes to the reduced DAT level in the axon terminals of p150^Glued^-deficient DAergic neurons. Further investigations will be required to determine how the PS-related mutations in p150^Glued^ detrimentally affect the structure and function of ER, exerting pathogenic impacts on the survival and function of the midbrain DAergic neurons.

While we focused the present study on the role of p150^Glued^ in midbrain DAergic neurons and PS-related parkinsonism, it remains to be determined the underlying pathological mechanisms of PS-related psychiatric symptoms such as depression and apathy, which appeared as an early clinical feature prior to parkinsonism^10–13^. Except for neuronal loss in the midbrain, the postmortem brains of PS patients also displayed variable degrees of neurodegeneration in other nuclei in basal ganglia, as well as in the hypothalamus, *locus coeruleus* (LC), periaqueductal gray matter, ventrolateral medulla, dorsal raphe nucleus, and pontine reticular formation^10–13^. In our cKO mice, deletion of p150^Glued^ was observed in ~100%, 46%, 2%, 99%, and 98% of TH^+^ cells in the midbrain, *locus coeruleus*, olfactory bulb, superior cervical ganglion, and adrenal medulla, respectively, suggesting differential efficiencies of Cre-mediated recombination in various populations of TH-expressing cells. On the one hand, this makes the cKO mice a valuable tool for studying the role of p150^Glued^ in the survival and function of DAergic neurons in the midbrain, sympathetic neurons in the superior cervical ganglion, and chromaffin cells in the adrenal medulla. On the other hand, this makes the cKO mice less suitable for studying the role of p150^Glued^ in the survival and function of TH-expressing neurons in the *locus coeruleus* and olfactory bulb. Future studies are needed to crossbreed the *Dctn1*^LoxP/^ mice with different Cre lines to continue exploring the role of p150^Glued^ in LC adrenergic neurons and other cell types. For example, the loss of serotonergic neurons in the medullary raphe and ventrolateral medulla may be responsible for the respiratory failure seen in PS patients^60^. It would be interesting to test this hypothesis with a selective deletion of p150^Glued^ in those serotonergic neurons using a suitable Cre line.

While TDP-43–positive cytoplasmic inclusions were often identified in the SN and other brain regions of PS patients^9,36,37^, we did not detect any apparent accumulation of TDP-43 in the cytosol of p150^Glued^-deficient DAergic neurons. This observation suggests the TDP-43 pathology might not contribute to the loss of midbrain DAergic neurons in the cKO mice during aging. Nonetheless, future studies would be needed to determine whether the TDP-43 pathology contributes to the degeneration of specific types of neurons with p150^Glued^ deficiency or mutations. In contrast, although the α-synuclein-containing Lewy bodies were rarely spotted in the postmortem brain of PS patients^10^, we observed somatic accumulation of α-synuclein and p-α-synuclein (Ser129) in the midbrain DAergic neurons of aged cKO mice. Besides, we found no apparent accumulation of α-synuclein in the soma of TH-negative neurons in the SNc, SNr, dorsal striatum, hippocampal CA1 area, and prefrontal cortex of aged cKO mice, indicating that the intercellular spread of α-synuclein pathology from midbrain DAergic neurons to other neurons in the neighboring and interconnected brain areas is a relatively rare event in our mouse model. Our findings are in line with previous studies that the abnormal accumulation of α-synuclein in cytosol and nucleus contributes to neurodegeneration^38,39^. However, it remains to determine how the deficiency of p150^Glued^ leads to α-synuclein pathology and how the accumulation of α-synuclein and the ER defects interplay within the p150^Glued^-deficient DAergic neurons.

In conclusion, to understand how the loss-of-function of p150^Glued^ protein contributes to PS-related parkinsonism and DAergic neuron loss, we genetically deleted the MTBDs-containing p150^Glued^ but kept the MTBD-lacking p135+ in the midbrain DAergic neurons of *Dctn1*^LoxP/LoxP^;*Th-Cre* cKO mice. The cKO mice developed progressive impairment of motor coordination and dysfunction/degeneration of DAergic neurons, including early-onset dendritic dystrophy, axonal swelling, DAT reduction, and dysregulated DA transmission, as well as late-onset neuronal death, axonal loss, and α-synuclein accumulation. We further revealed the impacts of p150^Glued^ deficiency on the ER in midbrain DAergic neurons, such as the reorganization of ER in dystrophic DAergic dendrites, the upregulation of ER tubule-shaping protein RTN3, the accumulation of DAT in reorganized ERs, the dysfunction of COPII-mediated ER export, the activation of UPR pathway, and the exacerbation of ER stress-induced cell death. Our studies raise the significance of defective ER structure and function in the degeneration of p150^Glued^-deficient DAergic neurons. The cKO mice may serve as a valuable animal model to further investigate the pathogenic mechanism and therapeutic targets of midbrain DAergic neuron degeneration in PS.

## Methods

### Animals

*Dctn1*^LoxP/^ mice (JAX, #032428) with two LoxP sites inserted in intron 1 and 4 of the *Dctn1* gene locus were generated in our previous work^22^. *Dctn1*^LoxP/^ mice were further crossbred with Cre recombinase (Cre) mouse lines to obtain *Dctn1*^LoxP/LoxP^;*Cre* mice, in which Cre recombinase-mediated deletion of *Dctn1* exons 2 to 4 abolished the expression of p150^Glued^ in the Cre-expressing cells^22^.

In this project, *Dctn1*^LoxP/^ mice were mated with *Th-Cre* mice (MMRRC, #029177-UCD), which have functional Cre expression in catecholamine DAergic and noradrenergic neurons^33^. This breeding strategy produced *Dctn1*^LoxP/LoxP^;*Th-Cre* mice [referred to as conditional knockout (cKO) mice], which had selective deletion of p150^Glued^ in midbrain DAergic neurons. The cKO mice and littermate controls were used for in vivo study.

In this project, *Dctn1*^LoxP/^ mice were also mated with *Cre/Esr1* mice (JAX, #004682) which have a tamoxifen-inducible form of Cre, capable of deleting floxed sequences in widespread cells or tissues^34^. This breeding strategy produced *Dctn1*^LoxP/LoxP^;*Cre/Esr1* mice [referred to as inducible knockout (iKO) mice], which had inducible deletion of p150^Glued^ expression after exposure to tamoxifen or 4-hydroxytamoxifen (4-OHT). The neonatal iKO pups and littermate controls were used for primary cell culture and in vitro study. Cohorts of 3-month-old Ctrl and iKO mice were intraperitoneally injected with tamoxifen (Sigma-Aldrich, solubilized in 100% sunflower seed oil) at the dosage of 100 mg/kg body weight for five consecutive days. One month after tamoxifen injection, midbrains of Ctrl and cKO mice were collected and used for ER microsomes preparation and biochemical assays.

All the mice were housed in a 12-h light/dark cycle and fed a regular diet *ad libitum*. All mouse work followed the guidelines approved by the Institutional Animal Care and Use Committees of the National Institute on Aging (No. 13-040) and Beijing Geriatric Hospital (No. BGH-2020-001).

### Genotyping

Genomic DNA was prepared from tail biopsy using DirectPCR Lysis Reagent (Viagen Biotech) and subjected to PCR amplification using specific sets of PCR primers for wild-type or floxed *Dctn1* gene (5′-CAGCTGCAAAGACCAGCAAA-3′ and 5′-CACACCACCTTCTTAGGCTTCA-3′) and *Cre* transgene (5′-CATTTGGGCCAGCTAAACAT-3′ and 5′-TGCATGATCTCCGGTATTGA-3′)^22^.

### Behavior tests

Body weight and motor function were repeated assessed on cohorts of male cKO mice (*n* = 16) and their littermate controls (*n* = 16) at 1, 3, 6, 12, and 18 months of age. Test performers were blinded to the genotypes of the mice.

Open-field test-Mice were placed in the open-field apparatus with infrared photobeam sensors. Locomotor activities (including ambulatory, rearing, and fine movement) and time spent in the center area (~40% of the total surface of the arena) of mice were measured by the Flex-Field Activity System (San Diego Instruments)^22^. Flex-Field software was used to trace and quantify mouse movement in the unit as the number of beam breaks per 30 min.

Rotarod test-Mice were placed onto a rotating rod with auto-acceleration from 0 rpm to 40 rpm for 1 min (San Diego Instruments)^22^. The length of time the mouse stayed on the rotating rod was recorded. Three measurements were taken for each animal during each test.

### Immunohistochemistry and light microscopy

Mice were sacrificed and transcardially perfused with 4% paraformaldehyde (PFA) in cold phosphate-buffered saline (PBS). Mouse brains, superior cervical ganglions, and adrenal glands were collected, post-fixed in 4% PFA/PBS solution overnight, submerged in 30% sucrose in PBS for at least 72 h, and sectioned at 40 μm thickness using CM1950 cryostat (Leica)^22^. Frozen sections were stained with antibodies specific to p150^Glued^ (amino acid 3–202 at the N-terminus of p150^Glued^, BD Biosciences, #610474, 1:200, recognizing p150^Glued^ but not p135+), p150^Glued^ & p135+ (amino acid 1266–1278 at the C-terminus of p150^Glued^, Abcam, #ab11806, 1:500, recognizing both p150^Glued^ and p135+), tyrosine hydroxylase (TH, Pel-Freez, #P40101-150, 1:2500; ImmunoStar, #22941, 1:500; Synaptic Systems, #213104, 1:500), dopamine transporter (DAT, Millipore, #MAB369, 1:500), vesicular monoamine transporter 2 (VMAT2, Synaptic Systems, #138302, 1:1000), glial fibrillary acidic protein (GFAP, Abcam, #ab7260, 1:1000), TAR DNA-binding protein 43 (TDP-43, Proteintech, #10782-2-AP, 1:500), α-synuclein (Santa Cruz, #sc-7011-R, 1:500; Santa Cruz, #sc-69977, 1:500), phosphorylated α-synuclein (Ser129) [p-α-synuclein (Ser129), Abcam, #ab51253, 1:500], neuronal nuclei (NeuN, Millipore, #ABN91, 1:500), synaptophysin (Millipore, #AB9272, 1:500), binding immunoglobulin protein (BiP, also referred to as GRP78, Abcam, #ab21685, 1:500), reticulon 3 (RTN3, Proteintech, #12055-2-AP, 1:500), 63 kDa cytoskeleton-linking membrane protein (CLIMP63, Proteintech, #16686-1-AP, 1:500), calnexin (Abcam, #ab22595, 1:500), protein disulfide isomerase (PDI, Proteintech, #11245-1-AP, 1:500), receptor binding cancer antigen expressed on SiSo cells (RCAS1, Cell Signaling Technology, #12290, 1:500), early endosome antigen 1 (EEA1, Cell Signaling Technology, #3288, 1:500), sequestosome 1 (SQSTM1, MBL, #PM066, 1:500), cathepsin D (R&D Systems, #AF1029, 1:500), ER-Golgi intermediate compartment 53 kDa protein (ERGIC53, Sigma-Aldrich, #E1031, 1:500), 130 kDa cis-Golgi matrix protein (GM130, BD Biosciences, #610822, 1:500), phosphorylated eukaryotic translation initiation factor 2α (Ser51) [p-eIF2α (Ser51), Abcam, #ab32157, 1:500], and phosphorylated inositol*-*requiring enzyme 1α (Ser724) [p-IRE1α (Ser724), Abcam, #ab48187, 1:500] as suggested by manufacturers. Alexa Fluor 488-, 546-, or 647-conjugated secondary antibody (Invitrogen, 1:500) was used to visualize the staining. Fluorescent images were captured using LSM 880 laser-scanning confocal microscope with Zen software (Zeiss) in conventional or Airyscan mode. As a high-resolution imaging modality, the Airyscan technology is reported to improve resolution 2-fold and signal-to-noise ratio 8-fold relative to the conventional confocal microscopy^61^. The paired images in all the figures were collected at the same gain and offset settings. Post-collection processing was applied uniformly to all paired images. The images were presented as a single optic layer after acquisition in z-series stack scans at 1.0 μm intervals from individual fields or displayed as maximum-intensity projection or three-dimensional (3D) reconstruction to represent confocal stacks.

### Stereology

Unbiased stereology was performed to estimate the number of midbrain DAergic neurons^62,63^. According to the mouse brain in stereotaxic coordinates, a series of 40-μm-thick coronal sections across the midbrain (every fourth section from Bregma −2.54 to −4.24 mm, ten sections per case) were stained with an antibody specific to TH (Pel-Freez, #P40101-150, 1:2500) and subsequently visualized with Vectastain Elite ABC Kit and DAB Kit (Vector Laboratories). Bright-field images were captured by Axio microscope Imager A1 (Zeiss). The number of TH-positive neurons was assessed using the optical fractionator function of Stereo Investigator 10 (MicroBrightField). Four or more mice were used per genotype at each time point. Counters were blinded to the genotypes of the samples. The sampling scheme was designed to have a coefficient of error <10% in order to obtain reliable results.

### Image analysis

For the quantitative assessment of various marker protein distributions, images were taken using identical settings and exported to ImageJ (NIH) for imaging analysis. Images were converted to an 8-bit color scale (fluorescence intensity from 0 to 255) by ImageJ (NIH). The areas of interest were first selected with Polygon or Freehand selection tools and then subjected to measurement by mean optical intensities or area fractions. The mean intensity for the background area was subtracted from the selected area to determine the net mean intensity. Data analyzers were blinded to the genotypes of the samples.

For quantitative analysis of the dystrophy of DAergic dendrites^32^, five tile images per animal (5 sections per animal and 1 tile image per section) from the SNr were taken with a ×40 lens. Dystrophic DAergic dendrites were defined as TH-positive neuritic varicosity ≥25 μm^2^. The number of dystrophic DAergic dendrites and the area of the SNr were quantified with ImageJ (NIH).

For quantitative analysis of the loss of DAergic axon terminals^32^, forty images per animal (10 sections per animal and 4 images per section) from the dorsal striatum were taken with a ×63 lens. The area fraction of DAergic axon terminals in each image was quantified with ImageJ (NIH).

For quantitative analysis of the swelling of DAergic axon terminals, five tile images per animal (5 sections per animal and 1 tile image per section) from the dorsal striatum were taken with a ×63 lens. Swollen DAergic axon terminals were defined as TH-positive neuritic varicosity ≥3 μm^2^. The number of swollen DAergic axon terminals and the dorsal striatum area were quantified with ImageJ (NIH).

### High-performance liquid chromatography (HPLC)

HPLC was performed to measure the content of DA and its metabolites in the striatum^32,63,64^. Mouse dorsal striatum was dissected, weighted, and homogenized in 500 μl buffer (0.1 N perchloric acid containing 100 μM EDTA) per 100 mg of tissue. After sonication and centrifugation, the supernatant was collected, frozen, and stored at −80 °C until assayed for DA, 3,4-dihydroxyphenylacetic acid (DOPAC), and 5-hydroxytryptamine (5-HT) by liquid chromatography with electrochemical detection. Briefly, the mobile-phase solution containing octanesulfonic acid as an ion-pairing agent was pumped isocratically through a reversed-phase liquid chromatographic column. DA, DOPAC, and 5-HT were quantified by the current produced after exposure of the eluate to a flow-through electrode set to oxidizing and then reducing potentials in series, with recordings from the last electrode reflecting reversibly oxidized species.

### Fast-scan cyclic voltammetry (FSCV)

To investigate the kinetics of DA release evoked by electrical stimulation, FSCV was performed in 400-μm-thick slices of the dorsal striatum^32,63^. Striatal slices were bathed in 32 °C oxygenated artificial cerebrospinal fluid [aCSF: 126 mM NaCl, 2.5 mM KCl, 1.2 mM NaH_2_PO_4_, 2.4 mM CaCl_2_, 1.2 mM MgCl_2_, 25 mM NaHCO_3_, 11 mM glucose, 20 mM 4-(2-hydroxyethyl)-1-piperazineethanesulfonic acid, 0.4 mM L-ascorbic acid]. Cylindrical carbon-fiber microelectrodes (50–100 μm of exposed fiber) were prepared with T650 fibers (6 μm diameter, Goodfellow) and inserted into a glass pipette. The carbon-fiber electrode was held at −0.4 V, and the potential was increased to 1.2 V and back at 400 V/s every 100 ms using a triangle waveform. DA release was evoked by rectangular, electrical pulse stimulation (100–600 μA, 0.6 ms per phase, biphasic) applied every 5 min. Data collection and analysis were performed using the Demon Voltammetry and Analysis software suite^65^. Ten cyclic voltammograms of charging currents were recorded as background before stimulation, and the average of these responses was subtracted from data collected during and after stimulation. Maximum amplitudes of extracellular DA transients were obtained from input/output function (I*/*O) curves. I*/*O curves were constructed by plotting stimulus current against the concentration of DA response amplitude over a range of stimulus intensities. The time constant of the slope decay (τ) was used for uptake kinetic analysis of evoked DA release. Following experiments, electrodes were calibrated using solutions of 1 and 10 μM DA in aCSF.

### Primary midbrain neuronal culture

Mouse primary midbrain neuronal cultures were prepared from newborn *Dctn1*^LoxP/LoxP^;*Cre/Esr1* pups and littermate controls on P0^22,62^. Briefly, midbrain tissues containing SNc and VTA were dissected and subjected to papain digestion (5 U/ml, Worthington Biochemicals) for 40 min at 37 °C. The digested tissue was carefully triturated into single cells using increasingly smaller pipette tips. The cells were then centrifuged at 250 × *g* for 5 min and resuspended in warm medium [Basal Medium Eagle (BME, Sigma-Aldrich), 1× N2/B27 supplement (the optimized serum-free supplement used to support the growth and viability of neurons, 100× stock, Invitrogen), 1× GlutaMax (100× stock, Invitrogen), 0.45% D-glucose (Sigma-Aldrich), 10 U/ml penicillin (Invitrogen), and 10 μg/ml streptomycin (Invitrogen)] supplemented with 5% heat-inactivated fetal bovine serum (FBS, Invitrogen). Dissociated midbrain neurons (~3 × 10^5^ cells per coverslip) were plated onto 12-mm round coverslips precoated with poly-D-lysine and laminin (BD Bioscience) in a 24-well plate and maintained at 37 °C in the 95% O_2_- and 5% CO_2_-humidified incubator. Twenty-four hours after seeding, the cultures were switched to the serum-free medium supplemented with 5 μM cytosine β-D-arabinofuranoside (Sigma-Aldrich) to suppress the proliferation of glia and 1 μM 4-OHT to induce CRE recombinase activity. From 5 days in vitro (DIV), culture medium was changed twice weekly.

### Assessment of DAergic neuron survival after chemical treatment or growth factor deprivation

For chemical treatment, the primary midbrain neurons at 14 DIV were exposed to vehicle (dimethyl sulfoxide, DMSO), 10 nM thapsigargin (an ER stress inducer, Sigma-Aldrich, #T9033), 100 nM GSK2606414 (a PERK inhibitor, MedChemExpress, #HY-18072)^66^, or 100 nM KIRA8 (an IRE1α inhibitor, MedChemExpress, #HY-114368)^67^ for 48 or 96 h. For growth factor deprivation, the primary midbrain neurons at 14 DIV were deprived of growth factor by lowering the content of N2/B27 supplement in the culture medium from 1× to 0.1× for 48 h. After chemical treatment or growth factor deprivation, neurons were fixed with 4% PFA/PBS and immunostained with TH antibody and secondary antibody. The number of TH-positive neurons on each coverslip was counted under the confocal microscope with a ×40 objective. Counters were blinded to the genotypes and treatments of the samples. The survival rate of DAergic neurons was calculated by dividing the number of TH-positive neurons on each coverslip by the number of TH-positive neurons on the control coverslip (neurons from *Dctn1*^LoxP/LoxP^ P0 pups treated with vehicle or cultured in normal medium)^62^.

### Primary fibroblast culture

Mouse fibroblast cultures were prepared from the dorsal skin of newborn *Dctn1*^LoxP/LoxP^;*Cre/Esr1* pups and littermate controls on postnatal day 0 (P0)^54^. Briefly, skin tissues were collected, rinsed in sterile PBS, and minced into small pieces. The minced tissues were triturated with 2 ml medium [DMEM (Invitrogen) supplemented with 10% FBS, 10 U/ml penicillin, and 10 μg/ml streptomycin], sparsely plated into a 10-cm culture dish, and maintained at 37 °C in the 95% O_2_- and 5% CO_2_-humidified incubator. After overnight (tissue fragments usually attach firmly to the dish), 8 ml fresh medium was added. After 3–4 days (fibroblasts grown out of tissue fragments usually start to undergo rapid proliferation), the medium was changed. At 7 DIV, fibroblasts were trypsinized with TrypLE (Invitrogen), passaged into new dishes, and treated with 1 μM 4-hydroxytamoxifen (4-OHT) to induce CRE recombinase activity. From 11 DIV on, the medium was changed every 3–4 days.

### Immunocytochemistry and light microscopy

Cultured cells on coverslips were fixed with 4% PFA in PBS for 15 min, permeabilized by 0.1% Triton X-100 for 5 min, and blocked in 10% normal donkey serum (Invitrogen) in PBS for 1 h at room temperature^54,62^. Cells were then labeled with primary antibodies against p150^Glued^ (BD Biosciences, #610474, 1:200, recognizing p150^Glued^ but not p135+), tyrosine hydroxylase (TH, Pel-Freez, #P40101-150, 1:2000), microtubule-associated protein 2 (MAP2, Abcam, #92434, 1:1000), binding immunoglobulin protein (BiP, also referred to as GRP78, Abcam, #ab21685, 1:500), and α-tubulin (Abcam, #ab89984, 1:1000) overnight at 4 °C in a humidified chamber. After three washes with PBS, secondary antibodies conjugated to Alexa Fluor (Invitrogen, 1:1000) were applied and incubated for 1 h at room temperature in the dark. After extensive washes, coverslips were mounted on glass slides with prolonged diamond antifade reagent containing DAPI (Invitrogen), and fluorescence signals were detected using LSM 880 laser-scanning confocal microscope (Zeiss). The paired images in all the figures were collected at the same acquisition settings, uniformly processed, presented as either a single optic layer or maximum-intensity projection of confocal stacks, and analyzed with ImageJ (NIH).

### Preparation of subcellular fractions

According to the manufacturer’s instructions, subcellular fractions were prepared using an endoplasmic reticulum isolation kit (Sigma-Aldrich). All procedures were performed at 4 °C. Mouse midbrains were isolated, cut into small pieces, and homogenized in four volumes of ice-cold Isotonic Extraction Buffer (10 mM HEPES, pH 7.8, 250 mM sucrose, 25 mM KCl, 1 mM EGTA, and 1× Protease and Phosphatase Inhibitor Cocktail) with Dounce homogenizer (12 strokes). Cultured cells were harvested, washed with ten volumes of PBS, and spun down at 600 × *g* for 5 min. The cell pellet was suspended and incubated in three volumes of ice-cold Hypotonic Extraction Buffer (10 mM HEPES, pH 7.8, 25 mM KCl, 1 mM EGTA, and 1× Protease and Phosphatase Inhibitor Cocktails) for 20 min to allow the cells to swell. Swollen cells were centrifuged at 600 × g for 5 min. The new cell pellet was homogenized in two volumes of ice-cold Isotonic Extraction Buffer with Dounce homogenizer (10 strokes). The homogenate (referred to as total lysate) from brain tissues or cultured cells was spun at 1000 × g for 10 min. The supernatant (S1) was collected, and the pellet (P1) was saved as crude nuclei fraction. S1 was centrifuged at 12,000 × *g* for 15 min. The supernatant (S2) was collected, and the pellet (P2) was saved as crude mitochondria fraction. S2 was further centrifuged at 100,000 × g for 60 min. The supernatant (S3) was collected as cytosol fraction, and the pellet (P3) was saved as ER microsomes fraction. Crude nuclei fraction, crude mitochondria fraction, and ER microsomes fraction were lysed in 1% SDS buffer. SDS was added to the total lysate and cytosol fraction to 1% final concentration. Equal amounts of protein from total lysate and each fraction were resolved in SDS-PAGE and applied to western blot analysis.

### Co-immunoprecipitation (co-IP)

Mouse brains or ER microsomes fraction of mouse brains were homogenized in IP buffer (50 mM Tris, pH 7.5, 150 mM NaCl, 10% glycerol, 50 mM NaF,10 mM glycerolphosphate, 2 mM EGTA, 2 mM EDTA, 1% NP-40, and 1× Protease and Phosphatase Inhibitor Cocktails) with Dounce homogenizer (10 strokes)^54^. Lysates were centrifuged at 15,000 × *g* for 15 min at 4 °C, and the supernatants were collected. The protein concentration of the lysates was measured and adjusted to 1 mg/ml. After pre-clearing with Protein G agarose (Thermo Fisher Scientific), the lysates were incubated with antibody-bound Protein G agarose for 1 h at 4 °C. After five washes of the agarose beads with IP buffer at 4 °C, the immune complexes were eluted with SDS sample buffer (Thermo Fisher Scientific) and examined by western blotting. The mouse-derived specific antibody against p150^Glued^ (BD Biosciences, #610474, 1:1000, recognizing p150^Glued^ but not p135+) and normal mouse IgG (Santa Cruz, #sc-2025) were used for co-IP.

### Western blotting

Cultured cells or mouse tissues were homogenized by sonication in ice-cold lysis buffer [50 mM Tris-HCl, 150 mM NaCl, 2 mM EDTA, pH 7.5, 1% SDS, and 1× Protease and Phosphatase Inhibitor Cocktails (Thermo Fisher Scientific)]^22,32^. Lysates were centrifuged at 15,000 × *g* for 15 min at 4 °C. The supernatants were collected and quantified for protein content using the bicinchoninic acid (BCA) assay kit (Thermo Fisher Scientific). Equal amounts of total protein were separated by NuPage 4–12% Bis-Tris gel using MES or MOPS running buffer (Thermo Fisher Scientific). The separated proteins were then transferred to nitrocellulose membranes using the Trans-Blot Turbo Transfer system (Bio-Rad) and incubated with specific primary antibodies. The antibodies used for western blot analysis included p150^Glued^ (BD Biosciences, #610474, 1:1000, recognizing p150^Glued^ but not p135+), p150^Glued^ & p135+ (Abcam, #ab11806, 1:1000, recognizing both p150^Glued^ and p135+), dynactin subunit 4 (DCTN4, Abcam, #ab170107, 1:1000), dynactin subunit p50 (BD Biosciences, #611002, 1:1000), dynactin subunit actin-related protein 1 (ARP1, Sigma-Aldrich, #A5601, 1:1000), tyrosine hydroxylase (TH, Sigma-Aldrich, #T1299, 1:1000), dopamine transporter (DAT, Millipore, #MAB369, 1:1000), VAMP (vesicle-associated membrane protein)-associated protein B (VAPB, Proteintech, #14477-1-AP, 1:1000), calnexin (Abcam, #ab22595, 1:1000), protein disulfide isomerase (PDI, Proteintech, #11245-1-AP, 1:1000), binding immunoglobulin protein (BiP, also referred to as GRP78, Abcam, #ab21685, 1:1000), endoplasmic reticulum-resident protein 72 (ERp72, Cell Signaling Technology, #5033, 1:1000), 63 kDa cytoskeleton-linking membrane protein (CLIMP63, Proteintech, #16686-1-AP, 1:1000), atlastin 1 (ATL1, Cell Signaling Technology, #12728, 1:1000), atlastin 2 (ATL2, Proteintech, #16688-1-AP, 1:1000), atlastin 3 (ATL3, Proteintech, #16921-1-AP, 1:1000), reticulon 1 (RTN1, Proteintech, #105048-1-AP, 1:1000), reticulon 3 (RTN3, Proteintech, #12055-2-AP, 1:1000), reticulon 4 (RTN4, Proteintech, #10950-1-AP, 1:1000), Sec13 [component of the coat protein complex II (COPII), Santa Cruz, #SC-514308, 1:1000], Sec23 (component of COPII, Thermo Fisher Scientific, #PA1-069A, 1:1000), Sec31 (component of COPII, BD Biosciences, #612351, 1:1000), nicastrin (Cell Signaling Technology, #3632, 1:1000), protein kinase-like endoplasmic reticulum kinase (PERK, Proteintech, #20582-1-AP, 1:1000), p-PERK (Thr982) (Thermo Fisher Scientific, #PA5-40294, 1:1000), eukaryotic translation initiation factor 2α (eIF2α, Cell Signaling Technology, #5324, 1:1000), phosphorylated eIF2α (Ser51) [p-eIF2α (Ser51), Abcam, #ab32157, 1:1000], activating transcription factor 4 (ATF4, Proteintech, #10835-1-AP, 1:1000), C/EBP-homologous protein (CHOP, Cell Signaling Technology, #2895, 1:1000), inositol-requiring enzyme 1α (IRE1α, Cell Signaling Technology, #3294, 1:1000), phosphorylated IRE1α (Ser724) [p-IRE1α (Ser724), Abcam, #ab48187, 1:1000], unspliced X-box binding protein 1 (unspliced XBP1, Proteintech, #25997-1-AP, 1:1000), spliced XBP1 (Proteintech, #24858-1-AP, 1:1000), stress-activated protein kinase/Jun-amino-terminal kinase (SAPK/JNK, Cell Signaling Technology, #9252, 1:1000), phosphorylated SAPK/JNK (Thr183/Tyr185) [p-SAPK/JNK (Thr183/Tyr185), Cell Signaling Technology, #4668, 1:1000], activating transcription factor (ATF6, Proteintech, #24169-1-AP, 1:1000), cleaved caspase-3 (Cell Signaling Technology, #9664, 1:1000), glyceraldehyde-3-phosphate dehydrogenase (GAPDH, Sigma-Adrich, #G9545, 1:1000), α-tubulin (Abcam, #ab7291, 1:5000), and β-actin (Sigma-Aldrich, #A1978, 1:5000). Protein signals were visualized by IRDye secondary antibodies and Odyssey system (LI-COR Biosciences) and quantified with ImageJ (NIH). Data analyzers were blinded to the genotypes of the samples.

### Statistical analysis

Statistical analysis was performed using GraphPad Prism 9 (GraphPad Software). Data were presented as mean ± SEM. Statistical significance was determined by comparing means of different groups using unpaired *t* test, one-way ANOVA with Tukey’s multiple comparisons test, two-way ANOVA with post-hoc Bonferroni test, or two-way ANOVA with Sidak’s multiple comparisons test. Not significant (ns), *p* ≥ 0.05; ^*^p < 0.05; ^**^p < 0.01; ^***^p < 0.001; ^****^p < 0.0001.

## Supplementary information

Supplementary Information

Supplementary Movie 1

Supplementary Movie 2

## Data Availability

The datasets generated and/or analyzed in this study are available upon request. Requests should be directed to Dr. Huaibin Cai caih@mail.nih.gov and Dr. Jia Yu jyu319@163.com.

## References

[1] Reck-Peterson SL, Redwine WB, Vale RD, Carter AP (2018). The cytoplasmic dynein transport machinery and its many cargoes. Nat. Rev. Mol. Cell Biol..

[2] Canty JT, Yildiz A (2020). Activation and regulation of cytoplasmic dynein. Trends Biochem. Sci..

[3] Radler MR, Suber A, Spiliotis ET (2020). Spatial control of membrane traffic in neuronal dendrites. Mol. Cell Neurosci..

[4] Cason SE, Holzbaur ELF (2022). Selective motor activation in organelle transport along axons. Nat. Rev. Mol. Cell Biol..

[5] Lipka J, Kuijpers M, Jaworski J, Hoogenraad CC (2013). Mutations in cytoplasmic dynein and its regulators cause malformations of cortical development and neurodegenerative diseases. Biochem. Soc. Trans..

[6] Cianfrocco MA, DeSantis ME, Leschziner AE, Reck-Peterson SL (2015). Mechanism and regulation of cytoplasmic dynein. Annu. Rev. Cell. Dev. Biol..

[7] Jaarsma D, Hoogenraad CC (2015). Cytoplasmic dynein and its regulatory proteins in Golgi pathology in nervous system disorders. Front. Neurosci..

[8] Puls I (2003). Mutant dynactin in motor neuron disease. Nat. Genet..

[9] Farrer MJ (2009). DCTN1 mutations in Perry syndrome. Nat. Genet..

[10] Konno T (2017). DCTN1-related neurodegeneration: Perry syndrome and beyond. Parkinsonism Relat. Disord..

[11] Mishima T (2018). Establishing diagnostic criteria for Perry syndrome. J. Neurol. Neurosurg. Psychiatry.

[12] Tsuboi Y, Mishima T, Fujioka S (2021). Perry disease: concept of a new disease and clinical diagnostic criteria. J. Mov. Disord..

[13] Dulski J (2021). Clinical, pathological and genetic characteristics of Perry disease-new cases and literature review. Eur. J. Neurol..

[14] Chung EJ (2014). Expansion of the clinicopathological and mutational spectrum of Perry syndrome. Parkinsonism Relat. Disord..

[15] Felicio AC (2014). In vivo dopaminergic and serotonergic dysfunction in DCTN1 gene mutation carriers. Mov. Disord..

[16] Mishima T (2016). Cytoplasmic aggregates of dynactin in iPSC-derived tyrosine hydroxylase-positive neurons from a patient with Perry syndrome. Parkinsonism Relat. Disord..

[17] Schroer TA (2004). Dynactin. Annu. Rev. Cell Dev. Biol..

[18] Dixit R, Levy JR, Tokito M, Ligon LA, Holzbaur EL (2008). Regulation of dynactin through the differential expression of p150Glued isoforms. J. Biol. Chem..

[19] Zhapparova ON (2009). Dynactin subunit p150Glued isoforms notable for differential interaction with microtubules. Traffic.

[20] Tokito MK, Howland DS, Lee VM, Holzbaur EL (1996). Functionally distinct isoforms of dynactin are expressed in human neurons. Mol. Biol. Cell..

[21] Hammesfahr B, Kollmar M (2012). Evolution of the eukaryotic dynactin complex, the activator of cytoplasmic dynein. BMC Evol. Biol..

[22] Yu J (2018). Genetic ablation of dynactin p150(Glued) in postnatal neurons causes preferential degeneration of spinal motor neurons in aged mice. Mol. Neurodegener..

[23] Lloyd TE (2012). The p150(Glued) CAP-Gly domain regulates initiation of retrograde transport at synaptic termini. Neuron.

[24] Moughamian AJ, Holzbaur EL (2012). Dynactin is required for transport initiation from the distal axon. Neuron.

[25] Lazarus JE, Moughamian AJ, Tokito MK, Holzbaur EL (2013). Dynactin subunit p150(Glued) is a neuron-specific anti-catastrophe factor. PLoS Biol..

[26] Levy JR (2006). A motor neuron disease-associated mutation in p150Glued perturbs dynactin function and induces protein aggregation. J. Cell Biol..

[27] Lai C (2007). The G59S mutation in p150(glued) causes dysfunction of dynactin in mice. J. Neurosci..

[28] Laird FM (2008). Motor neuron disease occurring in a mutant dynactin mouse model is characterized by defects in vesicular trafficking. J. Neurosci..

[29] Chevalier-Larsen ES, Wallace KE, Pennise CR, Holzbaur EL (2008). Lysosomal proliferation and distal degeneration in motor neurons expressing the G59S mutation in the p150Glued subunit of dynactin. Hum. Mol. Genet..

[30] Mishima T (2018). Behavioral defects in a DCTN1(G71A) transgenic mouse model of Perry syndrome. Neurosci. Lett..

[31] Deshimaru M (2021). Behavioral profile in a Dctn1(G71A) knock-in mouse model of Perry disease. Neurosci. Lett.

[32] Yu, J. et al. Selective expression of neurodegenerative diseases-related mutant p150Glued in midbrain dopaminergic neurons causes progressive degeneration of nigrostriatal pathway. *Ageing Neurodegener. Dis.* **2**, 10.20517/and.2022.07 (2022). 10.20517/and.2022.07 PMC12797871 41536567

[33] Gong S (2007). Targeting Cre recombinase to specific neuron populations with bacterial artificial chromosome constructs. J. Neurosci..

[34] Hayashi S, McMahon AP (2002). Efficient recombination in diverse tissues by a tamoxifen-inducible form of Cre: a tool for temporally regulated gene activation/inactivation in the mouse. Dev. Biol..

[35] Crittenden JR (2016). Striosome-dendron bouquets highlight a unique striatonigral circuit targeting dopamine-containing neurons. Proc. Natl. Acad. Sci. USA.

[36] Wider C (2009). Pallidonigral TDP-43 pathology in Perry syndrome. Parkinsonism Relat. Disord..

[37] Wider C (2010). Elucidating the genetics and pathology of Perry syndrome. J. Neurol. Sci..

[38] Cooper AA (2006). Alpha-synuclein blocks ER-Golgi traffic and Rab1 rescues neuron loss in Parkinson’s models. Science.

[39] Kontopoulos E, Parvin JD, Feany MB (2006). Alpha-synuclein acts in the nucleus to inhibit histone acetylation and promote neurotoxicity. Hum. Mol. Genet..

[40] Fujiwara H (2002). Alpha-Synuclein is phosphorylated in synucleinopathy lesions. Nat. Cell Biol..

[41] Uemura N, Uemura MT, Luk KC, Lee VM, Trojanowski JQ (2020). Cell-to-cell transmission of Tau and alpha-Synuclein. Trends Mol. Med..

[42] Wu Y (2017). Contacts between the endoplasmic reticulum and other membranes in neurons. Proc. Natl. Acad. Sci. USA.

[43] Sree, S., Parkkinen, I., Their, A., Airavaara, M. & Jokitalo, E. Morphological heterogeneity of the endoplasmic reticulum within neurons and its implications in neurodegeneration. *Cells* **10**, 10.3390/cells10050970 (2021). 10.3390/cells10050970 PMC8143122 33919188

[44] Westrate LM, Lee JE, Prinz WA, Voeltz GK (2015). Form follows function: the importance of endoplasmic reticulum shape. Annu. Rev. Biochem..

[45] Zhang H, Hu J (2016). Shaping the endoplasmic reticulum into a social network. Trends Cell Biol..

[46] Wang, N. & Rapoport, T. A. Reconstituting the reticular ER network - mechanistic implications and open questions. *J. Cell Sci.* **132**, 10.1242/jcs.227611 (2019). 10.1242/jcs.227611 30670475

[47] Sharoar MG (2016). Dysfunctional tubular endoplasmic reticulum constitutes a pathological feature of Alzheimer’s disease. Mol. Psychiatry.

[48] Sharoar MG, Zhou J, Benoit M, He W, Yan R (2021). Dynactin 6 deficiency enhances aging-associated dystrophic neurite formation in mouse brains. Neurobiol. Aging.

[49] Wang B (2018). The COPII cargo adapter SEC24C is essential for neuronal homeostasis. J. Clin. Invest..

[50] Tang BL (2021). Defects in early secretory pathway transport machinery components and neurodevelopmental disorders. Rev. Neurosci..

[51] Watson P, Forster R, Palmer KJ, Pepperkok R, Stephens DJ (2005). Coupling of ER exit to microtubules through direct interaction of COPII with dynactin. Nat. Cell Biol..

[52] Verissimo F, Halavatyi A, Pepperkok R, Weiss M (2015). A microtubule-independent role of p150glued in secretory cargo concentration at endoplasmic reticulum exit sites. J. Cell Sci..

[53] Dries DR, Yu G (2008). Assembly, maturation, and trafficking of the gamma-secretase complex in Alzheimer’s disease. Curr. Alzheimer Res..

[54] Cho HJ (2014). Leucine-rich repeat kinase 2 regulates Sec16A at ER exit sites to allow ER-Golgi export. EMBO J..

[55] Hetz C, Papa FR (2018). The unfolded protein response and cell fate control. Mol. Cell.

[56] Metcalf MG, Higuchi-Sanabria R, Garcia G, Tsui CK, Dillin A (2020). Beyond the cell factory: Homeostatic regulation of and by the UPR(ER). Sci. Adv..

[57] Collins MK, Perkins GR, Rodriguez-Tarduchy G, Nieto MA, Lopez-Rivas A (1994). Growth factors as survival factors: regulation of apoptosis. Bioessays.

[58] Sucic S (2011). The serotonin transporter is an exclusive client of the coat protein complex II (COPII) component SEC24C. J. Biol. Chem..

[59] Bu M, Farrer MJ, Khoshbouei H (2021). Dynamic control of the dopamine transporter in neurotransmission and homeostasis. NPJ Parkinsons Dis..

[60] Tsuboi Y (2008). Neurodegeneration involving putative respiratory neurons in Perry syndrome. Acta Neuropathol..

[61] Wu X, Hammer JA (2021). ZEISS airyscan: optimizing usage for fast, gentle, super-resolution imaging. Methods Mol. Biol..

[62] Liu G (2014). Aldehyde dehydrogenase 1 defines and protects a nigrostriatal dopaminergic neuron subpopulation. J. Clin. Invest..

[63] Liu G (2015). Selective expression of Parkinson’s disease-related Leucine-rich repeat kinase 2 G2019S missense mutation in midbrain dopaminergic neurons impairs dopamine release and dopaminergic gene expression. Hum. Mol. Genet..

[64] Holmes C, Eisenhofer G, Goldstein DS (1994). Improved assay for plasma dihydroxyphenylacetic acid and other catechols using high-performance liquid chromatography with electrochemical detection. J. Chromatogr. B Biomed. Appl..

[65] Yorgason JT, Espana RA, Jones SR (2011). Demon voltammetry and analysis software: analysis of cocaine-induced alterations in dopamine signaling using multiple kinetic measures. J. Neurosci. Methods.

[66] Axten JM (2012). Discovery of 7-methyl-5-(1-[3-(trifluoromethyl)phenyl]acetyl-2,3-dihydro-1H-indol-5-yl)-7H-pyrrolo[2,3-d]pyrimidin-4-amine (GSK2606414), a potent and selective first-in-class inhibitor of protein kinase R (PKR)-like endoplasmic reticulum kinase (PERK). J. Med. Chem..

[67] Morita S (2017). Targeting ABL-IRE1alpha signaling spares ER-stressed pancreatic beta cells to reverse autoimmune diabetes. Cell Metab..

