# Supplementary material for: Deficiency of Perry syndrome-associated p150Glued in midbrain dopaminergic neurons leads to progressive neurodegeneration and endoplasmic reticulum abnormalities
Source: NPJ Parkinsons Dis. 2023 Mar 7;9:35. doi: 10.1038/s41531-023-00478-0 (PMC9988887; doi:10.1038/s41531-023-00478-0)
Supplement: Supplementary file 1 — Supplementary Information [file 41531_2023_478_MOESM1_ESM.pdf]

Supplementary information

Supplementary figures

Supplementary Figure 1. P150^{Glued} deletion pattern in different TH-expressing cells of cKO mice.

Supplementary Figure 2. Inducible deletion of p150^{Glued} in cultured iKO neurons.

Supplementary Figure 3. No apparent accumulation of α-synuclein in the TH-negative neurons of cKO mice.

Supplementary Figure 4. The reorganized ER is the major organelle within the dystrophic DAergic dendrite of cKO mice.

Supplementary Figure 5. Both PERK inhibitor GSK2606414 and IRE1α inhibitor KIRA8 protect DAergic neurons against ER stress-induced cell death.

Supplementary movies

Supplementary Movie 1. 3D reconstruction of the ER and DAergic dendrites in the SNr of 6-month-old Ctrl mice (corresponding to Supplementary Figure 4h).

Supplementary Movie 2. 3D reconstruction of the ER and DAergic dendrites in the SNr of 6-month-old cKO mice (corresponding to Supplementary Figure 4h).

Uncropped western blots

Uncropped western blots shown in Figure 1b, f

Uncropped western blots shown in Figure 5f

Uncropped western blots shown in Figure 6e

Uncropped western blots shown in Figure 7g, h

Uncropped western blots shown in Figure 7j, l

Uncropped western blots shown in Figure 8e

Uncropped western blots shown in Supplementary Figure 2a

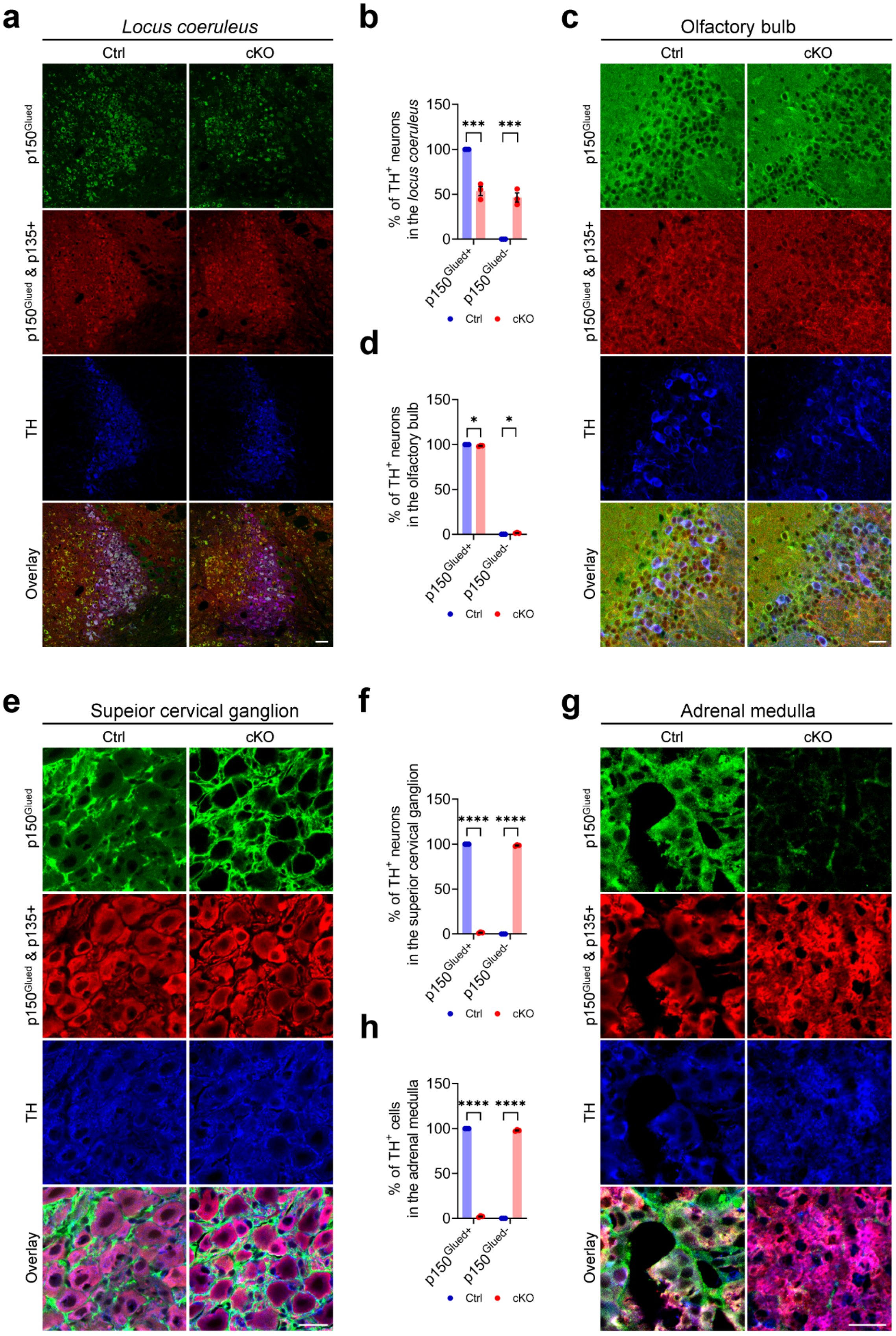
a
Locus coeruleus
Ctrl
cKO
p150Glued
p150Glued & p135+
TH
Overlay
b
% of TH+ neurons in the locus coeruleus
150
100
50
0
***
***
p150Glued+
p150Glued-
Ctrl
cKO
c
Olfactory bulb
d
% of TH+ neurons in the olfactory bulb
*
*
e
Supeior cervical ganglion
f
% of TH+ neurons in the superior cervical ganglion
****
****
g
Adrenal medulla
h
% of TH+ cells in the adrenal medulla

Supplementary Figure 1. P150^{Glued} deletion pattern in different TH-expressing cells of cKO mice. **a**, **c**, **e**, **g** Immunofluorescent images show the staining of p150^{Glued} (green), p150^{Glued} & p135+ (red), and TH (blue) in the *locus coeruleus* (**a**), olfactory bulb (**c**), superior cervical ganglion (**e**), and adrenal medulla (**g**) of 1-month-old Ctrl and cKO mice. Scale bar: 50 µm (**a**), 20 µm (**c**, **e**, **g**). **b**, **d**, **f**, **h** Bar graphs estimate the percentages of p150^{Glued}-positive and p150^{Glued}-negative TH$^+$ cells in the *locus coeruleus* (**b**), olfactory bulb (**d**), superior cervical ganglion (**f**), and adrenal medulla (**h**) of 1-month-old Ctrl and cKO (n = 3 animals per genotype and ≥ 3 sections per animal). Data were presented as mean ± SEM. Unpaired t-test, $^{***}p = 0.0008$ (b), $^{*}p = 0.0105$ (d), $^{****}p < 0.0001$ (f, h).

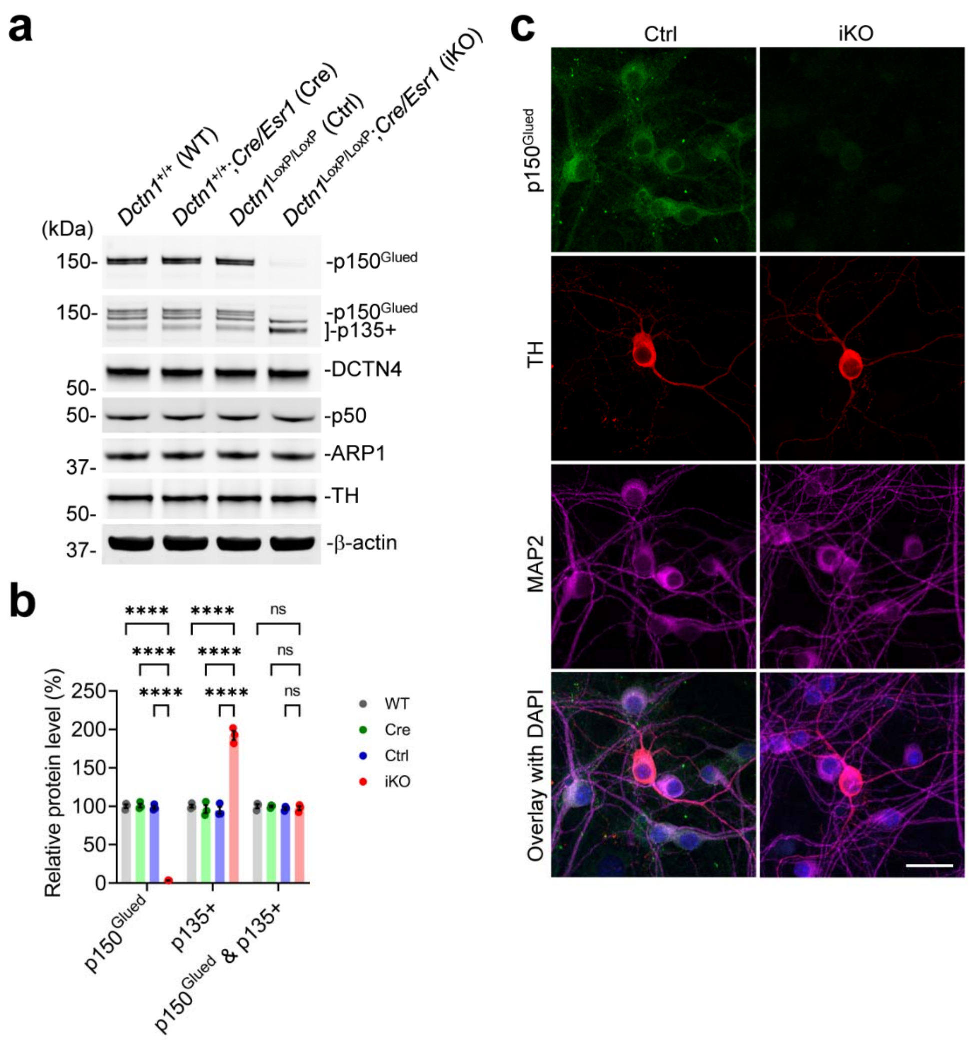

Supplementary Figure 2. Inducible deletion of p150^{Glued} in cultured iKO neurons. a Western blots show the expression of p150^{Glued}, p135+, DCTN4, p50, ARP1, and TH in the cultured midbrain neurons from WT (*Dctn1*$^{+/+}$), Cre (*Dctn1*$^{+/+}$;*Cre/Esr1*), Ctrl (*Dctn1*$^{\text{LoxP/LoxP}}$), and iKO (*Dctn1*$^{\text{LoxP/LoxP}}$;*Cre/Esr1*) mice at 14 days in vitro (DIV). At 1 DIV, 1 μM 4-OHT was added into the culture medium to induce CRE recombinase activity in cells. Actin and TH were used as the loading controls. **b** The Bar graph estimates the protein levels of p150^{Glued}, p135+, and p150^{Glued} & p135+ normalized with β-actin (n = 3 independent cultures). Data were presented as mean ± SEM. One-way ANOVA with Tukey's multiple comparisons test (the mean of each genotype was compared with the mean of every other genotype), $^{****}p < 0.0001$ (p150^{Glued}), $^{****}p < 0.0001$

(p135+). **c** Immunofluorescent images show the staining of p150^{Glued} (green), TH (red), MAP2 (magenta), and DAPI (blue) in the cultured Ctrl and iKO midbrain neurons at 14 DIV. Neurons were visualized by MAP2 staining. DAergic neurons were visualized by TH staining. Scale bar: 30 μm.

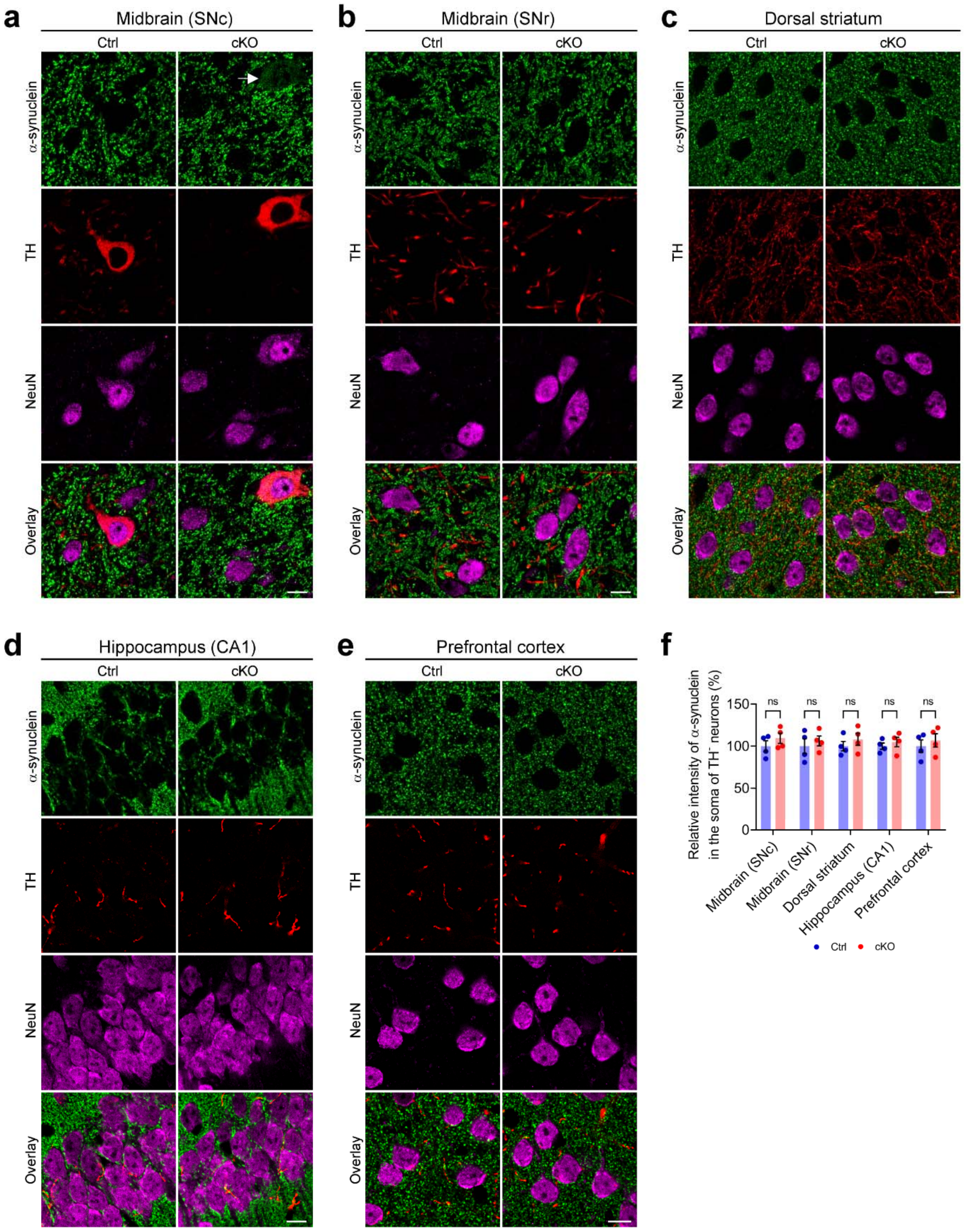

Supplementary Figure 3. No apparent accumulation of α-synuclein in the TH-negative neurons of cKO mice. a-e Immunofluorescent images show the staining of α-synuclein (green),

TH (red), and NeuN (magenta) in the SNc (**a**), SNr (**b**), dorsal striatum (**c**), hippocampal CA1 area (**d**), and prefrontal cortex (**e**) of 24-month-old Ctrl and cKO mice. The arrow points to the somatic accumulation of α-synuclein in the midbrain DAergic neurons of cKO mice. Scale bar: 10 μm. **f** The bar graph quantifies the staining intensity of α-synuclein in the soma of TH^- neurons in different brain regions of 24-month-old Ctrl and cKO mice (n = 4 animals per genotype and ≥ 30 neurons per animal). Data were presented as mean ± SEM. Unpaired t-test, ns ($p \geq 0.05$).

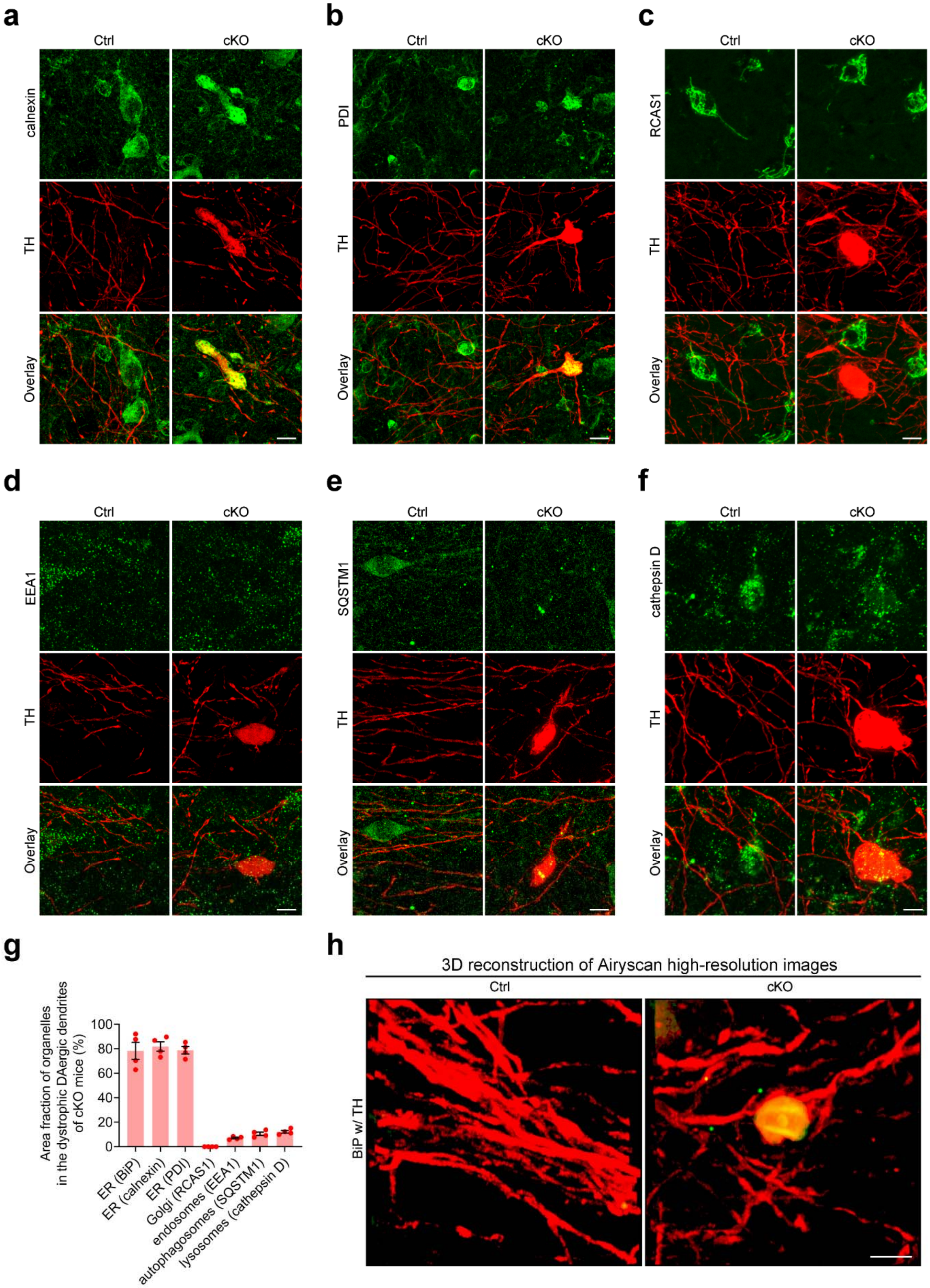

a
b
c
Ctrl
cKO
calnexin
PDI
RCAS1
TH
Overlay
d
e
f
EEA1
SQSTM1
cathepsin D
g
h
Area fraction of organelles in the dystrophic DAergic dendrites of cKO mice (%)
100
80
60
40
20
0
ER (BiP)
ER (calnexin)
ER (PDI)
Golgi (RCAS1)
endosomes (EEA1)
autophagosomes (SQSTM1)
lysosomes (cathepsin D)
3D reconstruction of Airyscan high-resolution images
BiP w/ TH

63

Supplementary Figure 4. The reorganized ER is the major organelle within the dystrophic DAergic dendrite of cKO mice. a Immunofluorescent images show the staining of calnexin (the ER marker, green) and TH (red) in the SNr of 6-month-old Ctrl and cKO mice. Scale bar: 10 μm. **b** Immunofluorescent images show the staining of PDI (the ER marker, green) and TH (red) in the SNr of 6-month-old Ctrl and cKO mice. Scale bar: 10 μm. **c** Immunofluorescent images show the staining of RCAS1 (the Golgi marker, green) and TH (red) in the SNr of 6-month-old Ctrl and cKO mice. Scale bar: 10 μm. **d** Immunofluorescent images show the staining of EEA1 (the endosome marker, green) and TH (red) in the SNr of 6-month-old Ctrl and cKO mice. Scale bar: 10 μm. **e** Immunofluorescent images show the staining of SQSTM1 (the autophagosome marker, green) and TH (red) in the SNr of 6-month-old Ctrl and cKO mice. Scale bar: 10 μm. **f** Immunofluorescent images show the staining of cathepsin D (the lysosome marker, green) and TH (red) in the SNr of Ctrl and cKO mice. Scale bar: 10 μm. **g** The bar graph estimates the area fractions of ER, Golgi, endosomes, autophagosomes, and lysosomes in the dystrophic DAergic dendrites of cKO mice (for each organelle marker, n = 4 animals and 3 sections per animal). **h** 3D reconstruction of Airyscan high-resolution images shows the ER (visualized by BiP staining, green) and DAergic dendrites (visualized by TH staining, red) in the SNr of 6-month-old Ctrl (see also Supplementary Movie 1) and cKO (see also Supplementary Movie 2) mice. Scale bar: 5 μm.

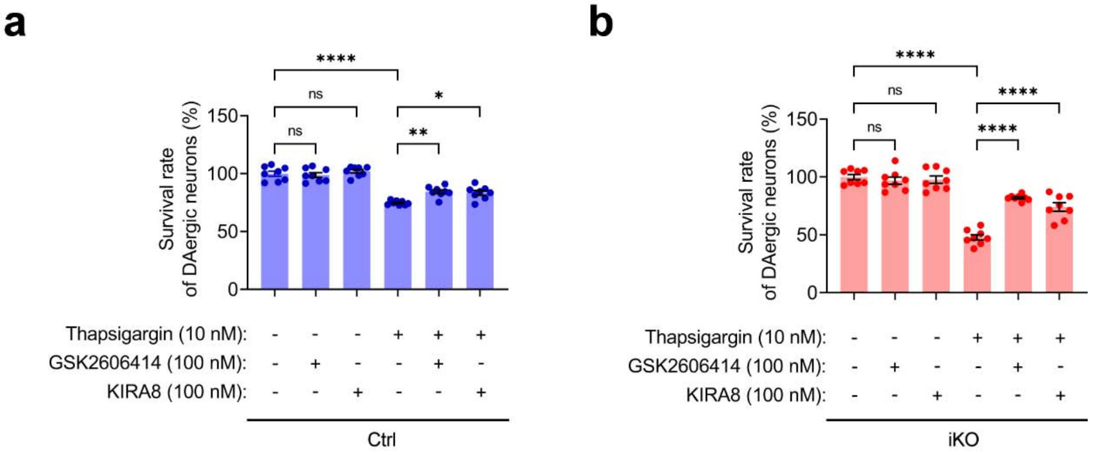

Supplementary Figure 5. Both PERK inhibitor GSK2606414 and IRE1α inhibitor KIRA8 protect DAergic neurons against ER stress-induced cell death. a The bar graph shows the survival rates of Ctrl midbrain DAergic neurons (14 DIV) treated with vehicle, 100 nM GSK2006414, or 100 nM KIRA8 in the presence of 0 or 10 nM thapsigargin (an ER stress inducer) for 48 hours (n = 8 coverslips per genotype per condition). Data were presented as mean ± SEM. One-way ANOVA with Tukey's multiple comparisons test, $^{*}p = 0.0124$, $^{**}p = 0.0038$, $^{****}p < 0.0001$. **b** The bar graph shows the survival rates of iKO midbrain DAergic neurons (14 DIV) treated with vehicle, 100 nM GSK2006414, or 100 nM KIRA8 in the presence of 0 or 10 nM thapsigargin for 48 hours (n = 8 coverslips per genotype per condition). Data were presented as mean ± SEM. One-way ANOVA with Tukey's multiple comparisons test, $^{****}p < 0.0001$.

Supplementary Movie 1. 3D reconstruction of the ER and DAergic dendrites in the SNr of 6-month-old Ctrl mice (corresponding to Supplementary Figure 4h). 3D reconstruction was generated from an Ariyscan high-resolution image stack. The ER was visualized by BiP (green) staining. DAergic dendrites were visualized by TH (red) staining.

Supplementary Movie 2. 3D reconstruction of the ER and DAergic dendrites in the SNr of 6-month-old cKO mice (corresponding to Supplementary Figure 4h). 3D reconstruction was generated from an Ariyscan high-resolution image stack. The ER was visualized by BiP (green) staining. DAergic dendrites were visualized by TH (red) staining.

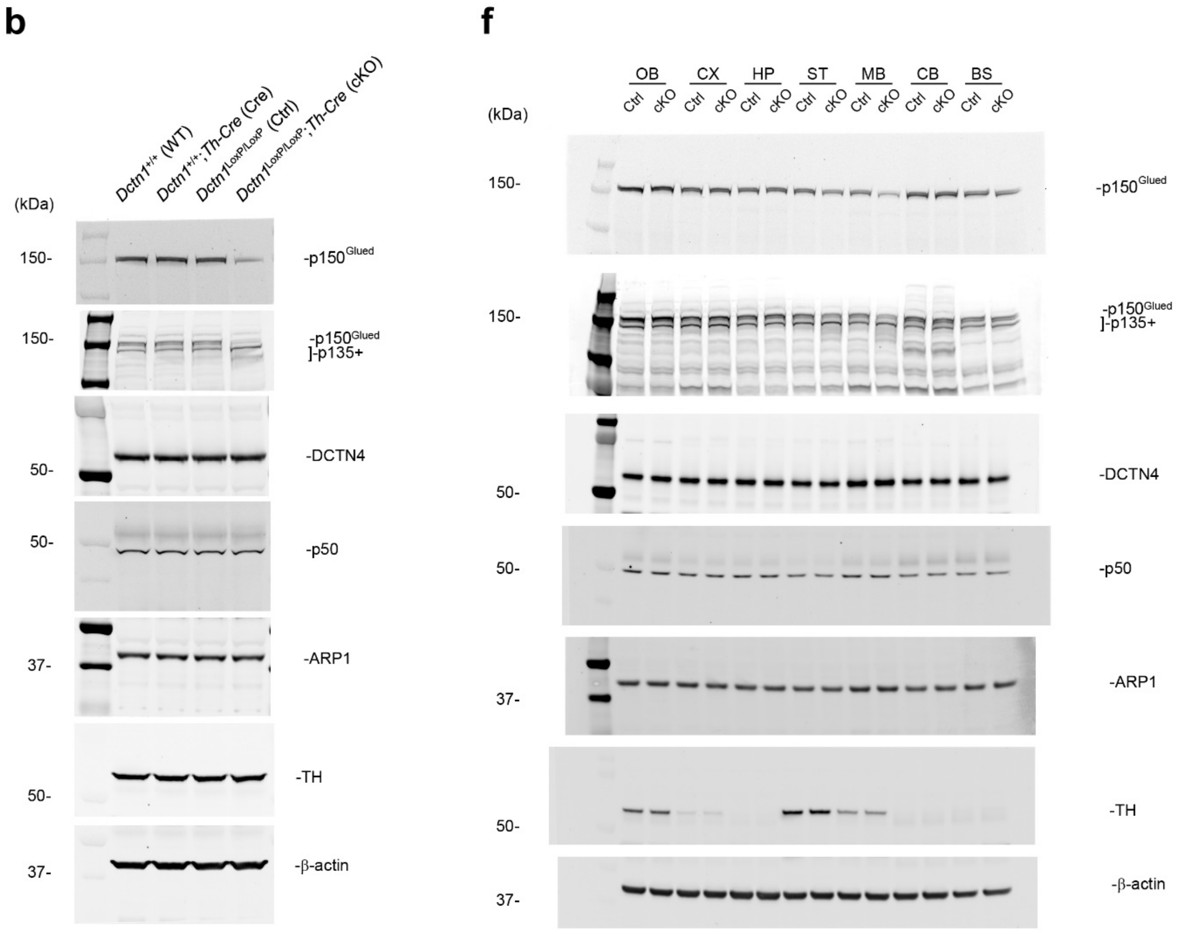

Uncropped western blots shown in Figure 1b, f

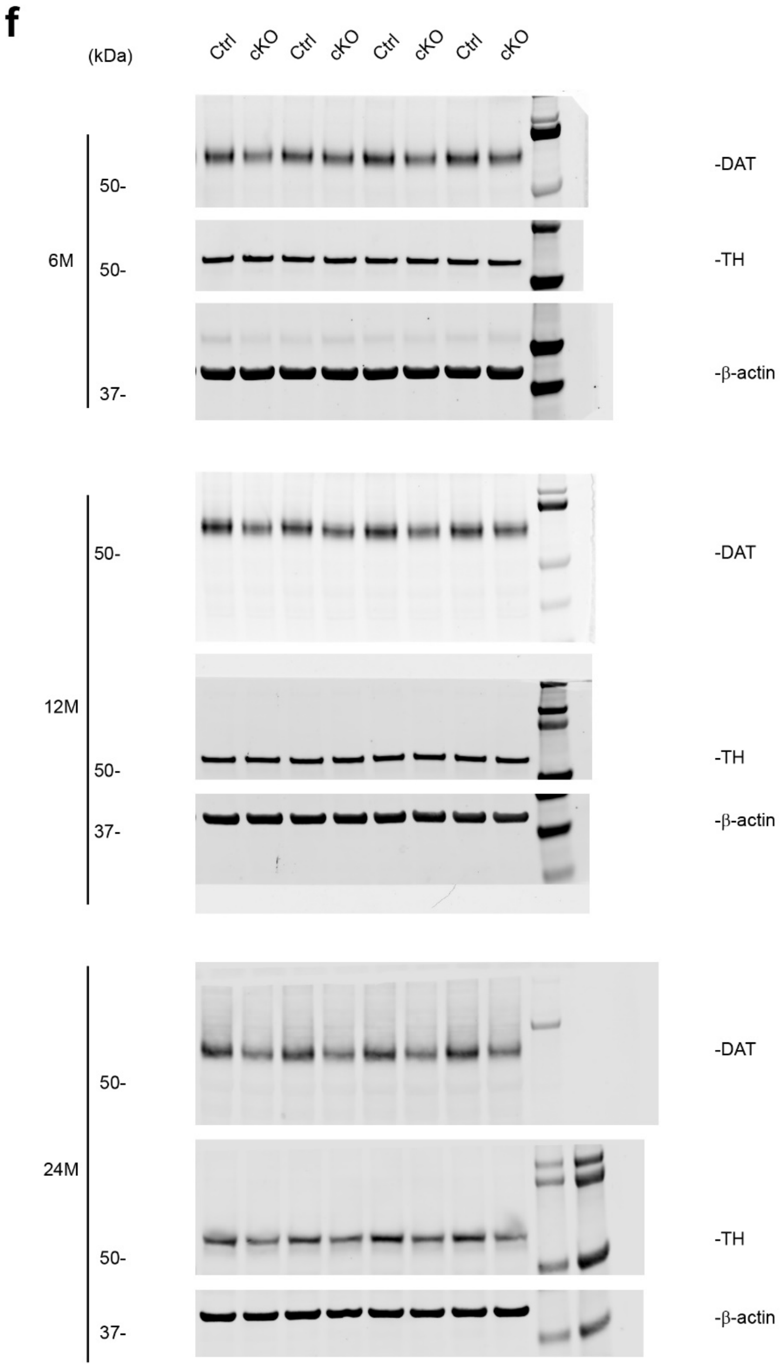

Uncropped western blots shown in Figure 5f

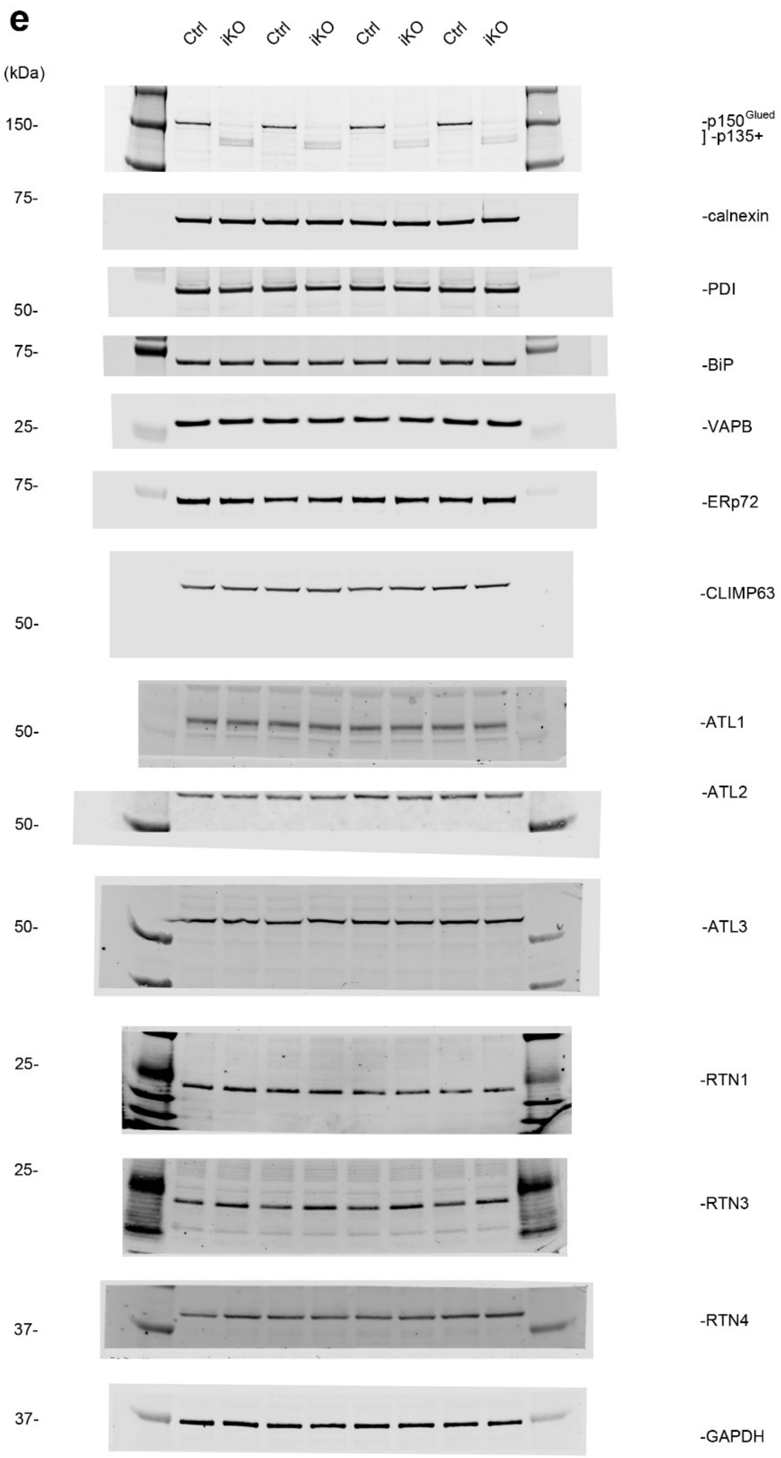

Uncropped western blots shown in Figure 6e

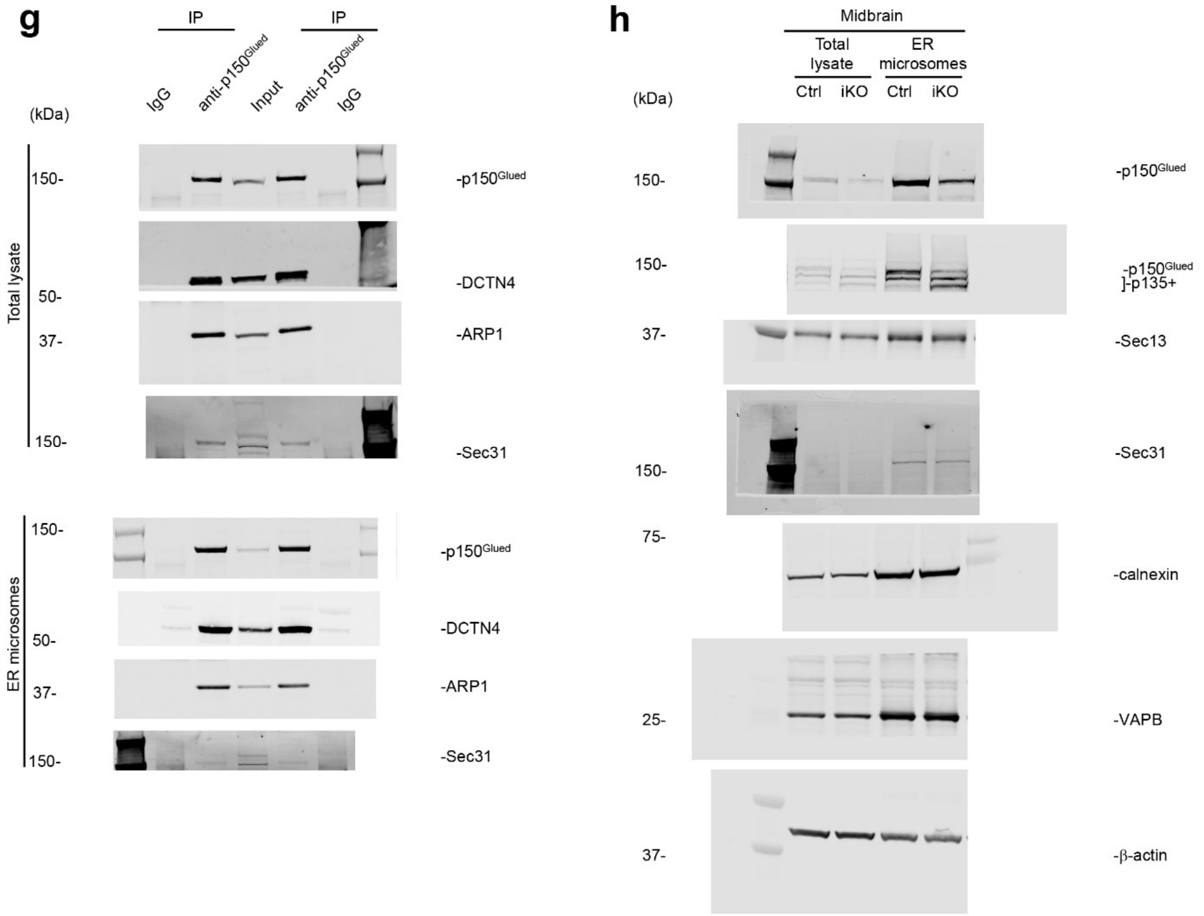

Uncropped western blots shown in Figure 7g, h

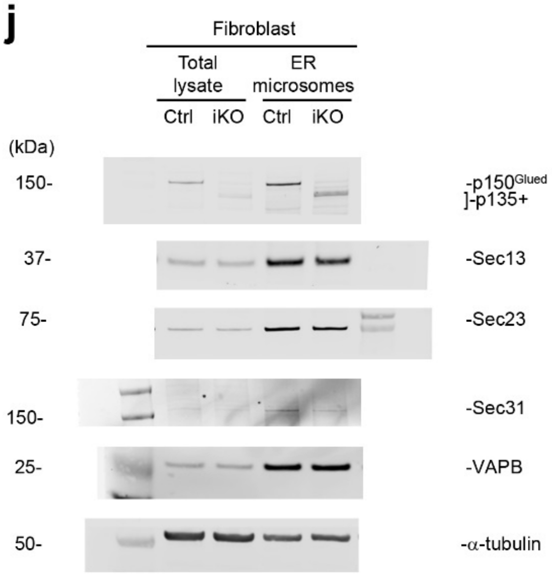

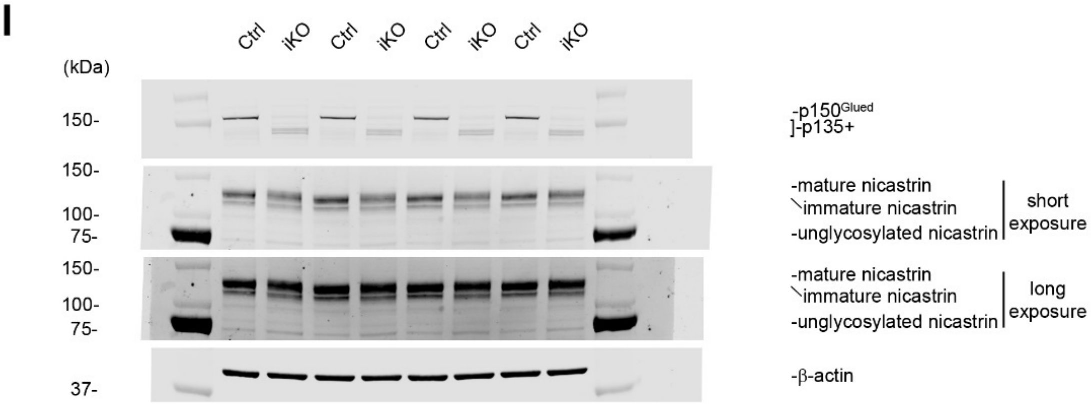

Uncropped western blots shown in Figure 7j, l

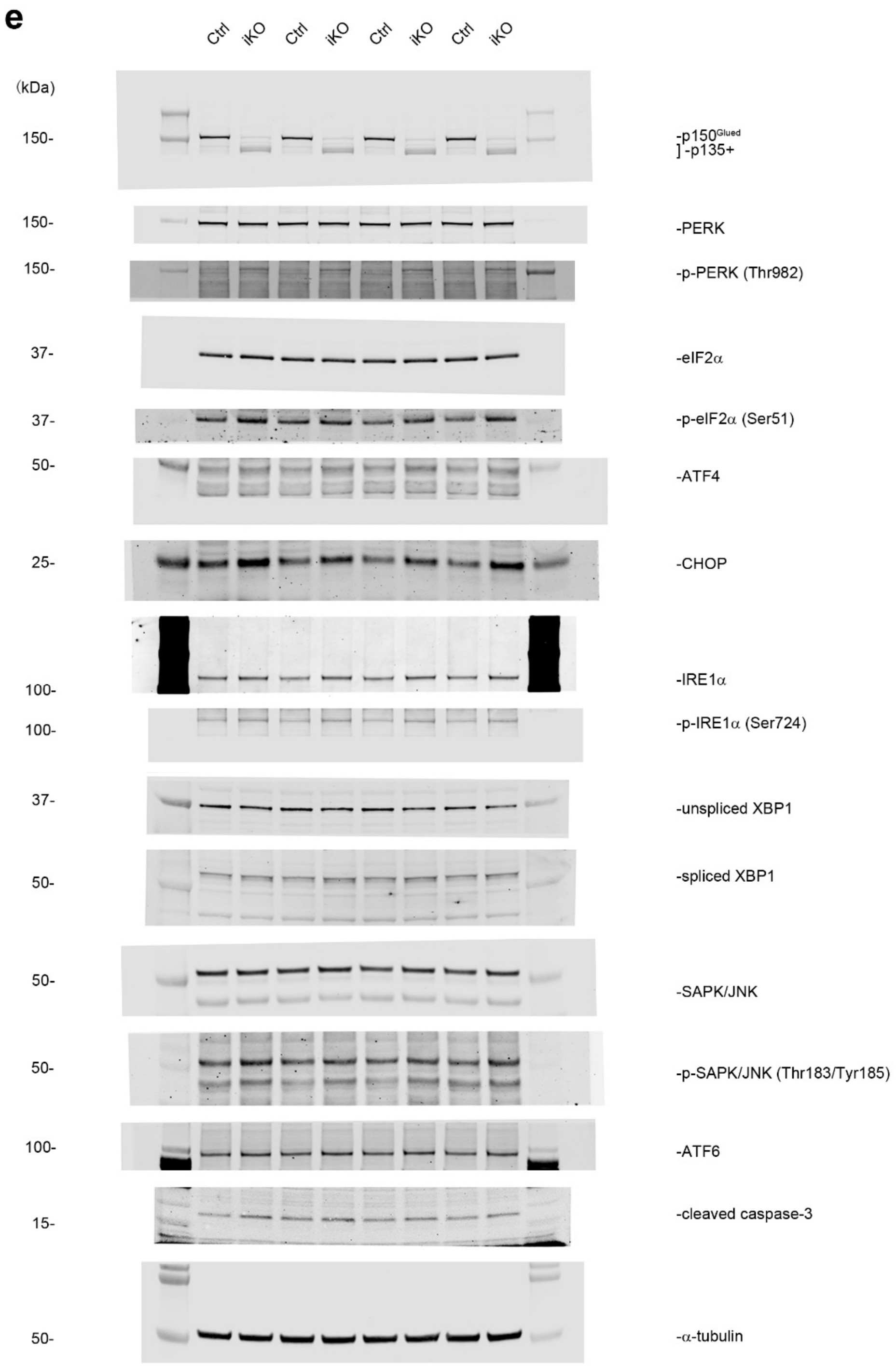

Uncropped western blots shown in Figure 8e

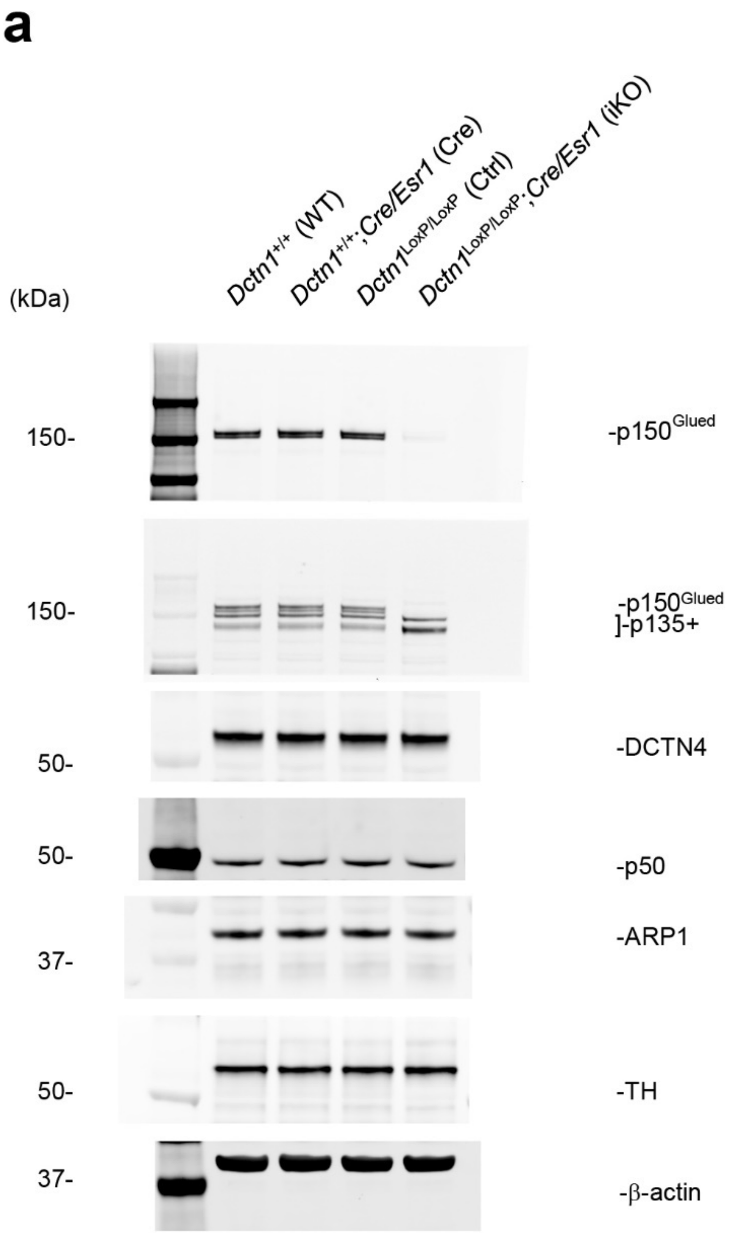

Uncropped western blots shown in Supplementary Figure 2a
